# Identification of Prognostic Markers in Cholangiocarcinoma Using Altered DNA Methylation and Gene Expression Profiles

**DOI:** 10.3389/fgene.2020.522125

**Published:** 2020-10-20

**Authors:** Nitish Kumar Mishra, Meng Niu, Siddesh Southekal, Prachi Bajpai, Amr Elkholy, Upender Manne, Chittibabu Guda

**Affiliations:** ^1^Department of Genetics, Cell Biology and Anatomy, University of Nebraska Medical Center, Omaha, NE, United States; ^2^Department of Pathology, University of Alabama at Birmingham, Birmingham, AL, United States

**Keywords:** cholangiocarcinoma, integrative analysis, differential expression, differential methylation, prognostic biomarker, TCGA, logistic regression

## Abstract

**Background:**

Cholangiocarcinoma (CCA) is a rare disease, but it is amongst the most lethal cancers with a median survival under 1 year. Variations in DNA methylation and gene expression have been extensively studied in other cancers for their role in pathogenesis and disease prognosis, but these studies are very limited in CCA. This study focusses on the identification of DNA methylation and gene expression prognostic biomarkers using multi-omics data of CCA tumors from The Cancer Genome Atlas (TCGA).

**Method:**

We have conducted a genome-wide analysis of differential DNA methylation and gene/miRNA expression using data from 36 CCA tumor and 9 normal samples from TCGA. The impact of DNA methylation in promoters and long-range distal enhancers on the regulation and expression of CCA-associated genes was examined using linear regression. Next, we conducted network analyses on genes which are regulated by DNA methylation as well as by miRNA. Finally, we performed Kaplan–Meier and Cox proportional hazards regression analyses in order to identify the role of selected methylation sites and specific genes and miRNAs in patient survival. We also performed real-time quantitative PCR (qPCR) to confirm the change in gene expression in CCA patients’ tumor and adjacent normal samples.

**Results:**

Altered DNA methylation was observed on 12,259 CpGs across all chromosomes, of which 78% were hypermethylated. We observed a strong negative relationship between promoter hypermethylation and corresponding gene expression in 92% of the CpGs. Differential expression analyses revealed altered expression patterns in 3,305 genes and 101 miRNAs. Finally, we identified 17 differentially methylated promoter CpGs, 72 differentially expressed genes, and two miRNAs that are likely associated with patient survival. Pathway analysis suggested that cell division, bile secretion, amino acid metabolism, PPAR signaling, hippo signaling were highly affected by gene expression and DNA methylation alterations. The qPCR analysis further confirmed that MDK, HNF1B, PACS1, and GLUD1 are differentially expressed in CCA.

**Conclusion:**

Based on the survival analysis, we conclude that DEPDC1, FUT4, MDK, PACS1, PIWIL4 genes, miR-22, miR-551b microRNAs, and cg27362525 and cg26597242 CpGs can strongly support their use as prognostic markers of CCA.

## Introduction

Cholangiocarcinoma (CCA), commonly known as the bile duct cancer, induces tumors in epithelial cells (cholangiocytes) that line the bile ducts (Lazaridis and Gores, 2005). The bile ducts are tubes from the liver and from the gall bladder that carry a fluid called bile into the small intestine, where it is used for digesting fat. CCA tumors may originate in the ducts that are inside (intrahepatic) or outside (extrahepatic) the liver. CCA is predominantly extrahepatic with only 10% of the cases having an intrahepatic origin. About 65% of CCA cases are diagnosed in seniors over the age of 65 with equal prevalence in both males and females. CCA prevalence has been steadily rising in the United States (from 0.044 per million in 1973 to 0.118 in 2012) (Saha et al., 2016) and also in other western countries (Khan et al., 2012). Because most patients are presented with advanced CCA at the time of diagnosis, despite chemotherapy, the survival for CCA patients is under 1 year (Valle et al., 2010).

Cholangiocarcinoma presents unique challenges for therapy due to its genetic heterogeneity. An earlier report (Chaisaingmongkol et al., 2017) has identified molecular subtypes of intrahepatic and extrahepatic cholangiocarcinoma in Asian populations. A recent classification of CCA in western populations proposed four possible molecular subtypes that differ in their DNA methylation, gene expression, copy number alteration, and mutation profiles (Farshidfar et al., 2017). In both studies, the most variable genes and DNA methylation sites were used for molecular subtyping; however, the global patterns of DNA methylation and gene/miRNA expression and their association with patient survival were not considered in these studies. The focus of our study is mainly on the identification of prognostic markers that are associated with survival using the western patient population that is represented in TCGA.

We have analyzed the patterns of DNA methylation, and gene and miRNA expression in CCA and control populations using TCGA data. Detailed correlative analyses between DNA methylation and gene expression; and gene expression and miRNA expression were also performed. Pathway and gene ontology (GO) enrichment analyses of differentially methylated genes (DMGs) and differentially expressed genes (DEGs) allowed us to gain insights into how alterations in DNA methylation and gene expression affect certain biological pathways to stimulate CCA progression. CCA-specific hypermethylated and hypomethylated distal enhancer and promoter probes and the well-known transcription factor binding motifs were examined to understand the site-specific transcription factors that are expected to be involved in carcinogenesis of CCA. We also carried out a network analysis of differentially expressed genes that are affected by promoter DNA methylation and miRNA expression to explore the role of the altered regulatory network in the CCA. Finally, we carried out an extensive analysis of CCA patient survival with respect to genetic markers, including differentially expressed genes, miRNAs, and differentially methylated promoter CpG sites. We also confirmed the change in the expression of selected genes in CCA compared to adjacent normal tissues using real-time qPCR.

## Materials and Methods

### DNA Methylation, RNA-Seq and miRNA-Seq Data

TCGA Firehose level-3 data on DNA methylation (Illumina HumanMethylation450 BeadArray), gene expression (IlluminaHiSeq RNASeqV2), and microRNA expression (IlluminaHiSeq, miRNAseq) were downloaded using Bioconductor tool, *RTCGAToolbox* (Samur, 2014). The DNA methylation level-3 data includes the β values for 485,577 CpG locations with annotations for chromosomes (UCSC hg19); HUGO Gene Nomenclature Committee (HGNC) gene symbols; and CpG coordinates (UCSC hg19). These β values were calculated as (M/M + U) range from 0 to 1, where M is the frequency of methylated allele and, U is the unmethylated allele frequency; higher β values suggested elevated methylation level. The gene expression data were acquired as single RSEM (RNA-seq by Expectation-Maximization) values for 20,531 HGNC genes. The miRNA-seq data have a single expression value for 1,046 miRNAs annotated with miRBase v16.

### Methylation Data Processing

To remove gender bias, β values of CpG probes that corresponded to X and Y chromosomes were discarded. We also discarded CpGs with missing β values in greater than 20% of the samples and used *k*-nearest neighbor-based imputation to assess the missing values with the imputeKNN module of R tool, *impute* data (Troyanskaya et al., 2001). Statistical analyses of DNA methylation of 45 samples (36 primary tumors and 9 normal samples) were performed at both the CpG site level and at the gene level. For distal enhancer analysis, probes that are 2 kb farther from the Transcription Start Sites (TSS) were used.

CpG probes were mapped to genes in six different sub-regions - TSS200 (the region from TSS to 200 bp upstream of TSS), TSS1500 (201–1,500 bp upstream from TSS), 5′UTR, 1st exon, gene body, and 3′UTR- and analyzed separately for each region (Supplementary Figure S1a). Similarly, DNA methylation in the CpG island regions including shore (0–2 kb from CpG islands), shelf (2–4 kb after CpG islands), and open sea regions (CpG sites anywhere in the genome without a specific designation) were also examined (Supplementary Figure S1b). We created ‘gene region collapsed data’ for dimensionality reduction of the methylation data on regions that are most appropriate for gene function. R version 3.4.2 (R Development Core Team, 2015) was used to perform the analyses. The β values for every sub-region were summarized utilizing the median, i.e., if a gene has two or more CpG sites in the same sub-region then the median of β values (by using R function ‘*aggregate*’) was used.

### RNA-Seq and miRNA-Seq Data Processing

The level-3 RNA-seq data contain expression values for each gene obtained by normalizing the expression values for each gene in every samples. Briefly, these values were derived by mapping RNA-seq reads with MapSplice software to the human reference genome and quantifying transcripts with RSEM (Li and Dewey, 2011). TCGA level-3 has different types of RSEM output data; the expected read count data was used in this analysis. Level-3 miRNA-seq data contains raw read count for each miRBase v16 miRNA, derived from the exact mapping of miRNA-seq reads with BWA-MEM, and quantified with ShortStack (Axtell, 2013). We purged all samples and genes or miRNAs from the downstream analysis that have missing expression values (NA) for over 20% of the samples, genes or miRNAs.

### Batch Effect and Unsupervised Analysis of Data

Using Mbatch (TCGA, 2010), Principal Component Analysis (PCA) was performed to identify potential batch effects among the samples for DNA methylation, gene expression, and miRNA expression data. The unsupervised clustering of epigenome and transcriptome data were performed by PCA in R using *princomp* function. Pearson correlation matrix, Manhattan distance matrix and heatmaps of the tumor and normal samples were generated using *ggplot2* in R. Three dimensional PCA plot was generated with R packages, *ggplot2*, and *scatterplot3d* (Wickham, 2016).

### Genome-Wide Analysis of Differentially Methylated CpG Probes

Methylation probes which mapped in the repeated regions of chromosomes and having a known SNP within 10 bp of interrogated CpG locations were also removed as recommended by other TCGA studies (Cancer Genome Atlas Network, 2012; Cancer Genome Atlas Research Network, 2014). Next, we normalized β values by using the Beta Mixed Integer-quantile normalization (BMIQ) (Teschendorff et al., 2013) method to correcting the bias of type II probes in Illumina HumanMethylation450K BeadChip data by utilizing the Bioconductor tool, *ChAMP* (Morris et al., 2014). R package, *limma* (Ritchie et al., 2015) was used for supervised differential methylation analysis. For a given CpG site to be considered differentially methylated, the difference between the median DNA methylation level in the primary tumor and normal samples must be at least 0.2 (Δβ ≥ 0.2) and the BH adjusted *p*-value ≤ 0.005. *Limma* was also used on summarized methylation data for differential CpG island analysis. A circular plot of differentially methylated CpGs (dm-CpGs) with differential methylation frequencies in 10 Mb sliding window for each chromosome was generated by using R tool, *gtrellis* (Gu et al., 2016). Then differential methylation frequency per mega base pair (Mb) was determined for each chromosome by computing the overall number of dm-CpGs for each chromosome and dividing by the length of the respective chromosome (Mb). Similarly, for each chromosome, hypermethylation and hypomethylation frequencies were also calculated. A particular chromosome considered predominantly hypermethylated if the ratio between hypermethylation to hypomethylation frequencies is ≥1.5. Similarly, if a chromosome has hypomethylation to hypermethylation frequency ratio is ≥1.5 considered as predominately hypomethylated.

### Genome-Wide Analysis of Differentially Methylated Regions (DMRs)

A genomic region containing at least two dm-CpGs was considered as a differentially methylated region (DMR). A Bioconductor tool, *DMRcate* (TCGA, 2010) was used to analyze genome-wide DMRs. For regulatory region analysis, we mapped DMRs in super-enhancers (Pott and Lieb, 2015), Vista enhancers (Visel et al., 2007), and DNase hypersensitive sites (Consortium, 2012), which are known regulatory regions. Super-enhancers are genomic regions containing groups of transcriptionally active putative enhancers in close proximity. VISTA enhancers are individual experimentally validated human or mouse non-coding sequences with gene enhancer activity. While DNase hypersensitive regions are specific regions of the genome, where chromatin attains open structure, i.e., euchromatin, making it accessible for transcription. We mapped DMRs against these three types of regulatory regions and DMRs that show at least a 10% overlap with these regions are considered as active regulatory regions.

### Differential Gene Expression Analysis

For differential gene expression analysis, 36 primary CCA and 9 normal samples expected counts’ data were used. We preprocessed the data to remove all genes that have low (≤1 count per million, CPM) or missing values in over 20% of the samples. After preprocessing, Bioconductor tools, *edgeR* (Robinson et al., 2010) and *DESeq2* (Love et al., 2014) were used for differential gene expression analysis using an absolute logFC cutoff at 1.5 and both the raw *p*-value and Benjamini–Hochberg (BH) (Benjamini and Hochberg, 1995) adjusted *p*-value cutoffs at 0.01. For differential miRNA expression analysis, we used level-3 raw read counts data in *edgeR*, logFC ≥ 1, and a BH corrected *p*-value of 0.01.

### Classification of the Tumor and Normal Samples

We used logistic regression models by applying linear models (*lm*) function in R to categorize tumor and normal samples using gene expression, miRNA expression, and DNA methylation data. R package *ROCR* (Sing et al., 2005) was used to evaluate the performance of *lm* for each gene, miRNA, and CpGs by using the area under the curve (AUC). R tool *ROCR* was used for the receiver operating characteristic (ROC) curve plot. We used only those genes, miRNAs, or CpGs that have an AUC ≥ 0.70 for further analysis.

### Association Between DNA Methylation and Gene Expression

For association analysis, a total of 36 primary tumor samples that have both DNA methylation and gene expression data were examined. R package *eMap* (Sun, 2010) was used for the linear regression-based correlation analysis between DNA methylation and gene expression. Methylation and expression levels of genes were verified for non-zero association using Pearson’s correlation, i.e., excluding all those which have a correlation value of zero. For further analysis, probes within 100 kb from the TSS of a gene in either direction were used, and an association was taken into account as significant only if the Bonferroni corrected *p*-value was under 0.05. R package *quantsmooth* (Oosting et al., 2014) was used to visualize the genome-wide association between DNA methylation and gene expression.

The median methylation of CpGs in the ‘gene region collapsed’ regions, and gene expression of corresponding genes was assessed for non-zero correlation using Spearman correlation (R function *cor.test*). Correlation between DNA methylation and gene expression was regarded as noteworthy if the raw *p*-value and BH-corrected *p*-value were both below 0.05.

### Enrichment Analysis

Bioconductor package *clusterProfiler* (Yu et al., 2012) and the desktop version of GSEA (Subramanian et al., 2005) were used for enrichment analysis of DEGs in CCA. Entrez gene ids were used with a BH multiple adjustment threshold of 0.05 with a minimum and maximum of five and 500 genes, respectively, for each pathway. KEGG, Reactome, Biocarta, and PID were utilized for the pathway enrichment analysis. Another tool, ‘camera’ in *limma*, was used for pathway enrichment analysis by selecting only overlapping pathways. We used ‘*gometh*’ module of the Bioconductor tool *missMethyl* (Phipson et al., 2016) for the pathway enrichment analysis of dm-CpGs. Genes associated with dmCpGs in the IlluminaHuman450K BeadChip with Δβ ≥ 0.2 were acquired from the annotation package, IlluminaHumanMethylation450kanno.ilmn12.hg19. Every GO and KEGG terms were examined using ‘*gometh*’, and false discovery rates (FDR) were calculated by using the BH method.

### Regulatory Element Landscape and Transcription Factor Analysis

For regulatory element analysis, we have used the Bioconductor tool, Enhancer Linking by Methylation/Expression Relationship (*ELMER*) (Yao et al., 2015). *ELMER* uses ENCODE/REMC, ChromHMM, and FANTOM5 genomic regions for annotating enhancer regions. We used level-3 data of tumor specimens that had both DNA methylation and gene expression values. Using *ELMER*, we determined the distal enhancer (>2.0 kb away from known TSS) and promoter (within 2 Kb from known TSS) probes and correlated their DNA methylation states with the expression of genes in close proximity to identify transcriptional targets. First, we selected distal enhancer and promoter probes and used one-tailed *t*-test to identify hypermethylated and hypomethylated CpG probes. In the second step, Pearson’s correlation among the differentially methylated distal enhancer probe and 10 nearby up- and downward genes’ expression values were calculated to determine the putative target gene and distal enhancer probe pair. To achieve a high confidence correlation between distal enhancer probes and gene expression data, we used 10,000 permutations. For promoter analysis, we used the nearest single gene and calculated correlation to determine the relationship. Next, FIMO (find individual motif occurrences) tool (Grant et al., 2011) was used to locate the enriched TF binding motifs for differentially methylated distal enhancer or promoter probes, which are significantly correlated with a putative target gene, with a *p*-value cutoff of 1e^–4^. FIMO was used to scan for enriched TF binding motifs within ±100 bp region in the neighborhood of the individual probe utilizing position weight matrices (PWMs) of the human TF motif database, JASPAR-Core (Mathelier et al., 2014) and Factorbook (Wang et al., 2013). Finally, a list of upstream master regulator TFs corresponding to each of the enriched TF binding motifs whose expression has been associated with the DNA methylation of the TF binding motif region was determined by using *ELMER*. For each motif, the average DNA methylation of all distal enhancer/promoter probes within ±100 bp of a motif occurrence region was computed and correlated with the 1,982 known human TFs expression (Ravasi et al., 2010). Then, for each motif-gene pair, we made two separate groups of samples: *M* group (20% of the specimens with the highest average DNA methylation for a motif) and *U* group (20% of the specimens with the lowest average DNA methylation for a motif). We used the Wilcoxon rank-sum test to test the null hypothesis that *M* group had greater or equal overall gene expression than the *U* group, for each candidate motif-TF pair. All TFs were ranked by the -log10 (*P*), and the ones that falls within the upper 5% of this ranking were considered potential upstream master regulators.

### Network Analysis for Genes Under Multiple Regulatory Control (GMRCs)

Molecular interaction networks (interactome) were used to understand the interaction between genomic and epigenomic loci associated with CCA. Gene expression correlated with both methylation and miRNA expression were used to build the gene network of interacting molecules using BisoGenet (Martin et al., 2010) plugin in Cytoscape (Shannon et al., 2003). The network was constructed by using the molecular interactions from various biological interaction databases that included BIND, BioGRID, DIP, HPRD, INTACT, MINT, ENCODE, and Microcoms. Topological parameters of the network were calculated using the CentiScaPe plugin in Cytoscape (Scardoni et al., 2009). Genes that are part of more than one association study, i.e., listed in more than one QTL analysis (DNA methylation, miRNA) were treated as genes under multiple regulatory controls (GMRCs). GMRCs and their first interactive neighbors were used to construct the network and perform a topological analysis using neighborhood connectivity and node stress parameters from CentiScaPe, to estimate the average rank (AR) score for each node. The AR score was decided by an average rank of the nodes with respect to both indices, i.e., node stress and neighborhood connectivity. The nodes with AR score cutoff ≤10 were considered as key regulatory hubs. The significance of pathways associated with sub-networks of hub nodes was decided by Fisher’s exact test *p*-value in Ingenuity Pathways Analysis (IPA) for a query gene-based search for pathway enrichment (Kramer et al., 2014).

### Survival Analysis

To uncover the role of differentially expressed genes/miRNA in the survival of patients, CCA patients were segregated into two groups, namely high and low, using the median expression of genes as a cut-off value. To evaluate DNA methylation, we used 0.5 and 0.3 as β value cutoff for the high and the low groups. Only those promoter CpGs which were within 1000 bp downstream or 500 bp upstream of the TSS of the genes were used for survival analysis (Cooper et al., 2006; Whitfield et al., 2012). The R tool, *survival* was used for survival analysis, and the Kaplan–Meier survival curve plot was generated for all analyses. In addition, a log-rank or Mantel–Haenszel test was conducted to observe the difference between survival curves of two groups and to calculate the *p*-value.

### Sample Procurement and Real-Time Quantitative PCR (qPCR) Analysis

Twenty-one snap-frozen cholangiocarcinoma (CCA) specimens, including seven samples with tumor-adjacent normal tissues, were collected from the University of Alabama at Birmingham (UAB) Tissue Biorepository, after obtaining approval from the Institutional Review Board. All samples were stored in the Invitrogen RNA*later*-ICE stabilization solution (Thermo Fisher Scientific, Waltham, MA, United States) until processed for RNA extraction and real-time quantitative PCR (qPCR).

Total RNA was isolated from CCA and normal specimens with TRIzol reagent using the manufacturer’s instructions (Invitrogen, Carlsbad, CA, United States). Total RNA (2 μg) was reverse transcribed using high-capacity cDNA reverse transcription kits with RNase inhibitor (Applied Biosystems, Thermo Fisher Scientific, Waltham, MA, United States). The cDNA (10-ng) samples were used for validation and quantification of four genes (MDK, HNF1B, PACS1, and GLUD1) using PowerUp SYBR green master mix (Applied Biosystems, Thermo Fisher Scientific, Waltham, MA, United States) on an ABI real-time PCR machine and analyzed with Quant-studio real-time PCR software (Applied Biosystems, Thermo Fisher Scientific, Waltham, MA, United States). The Student’s *t*-test with two-tailed distribution was used to calculate the p-values for the statistical significance.

## Results

TCGA level-3 DNA methylation gene expression and miRNA expression data downloaded by using the Bioconductor tool, *RTCGAToolbox* (Samur, 2014), and thoroughly carried out data cleaning, batch effect testing, global unsupervised analyses and comprehensive single and integrative analyses on DNA methylation, mRNA and miRNA expression datasets. To recognize the functional relevance and consequences of the differentially expressed genes in CCA, we also completed downstream analyses using clustering and pathway enrichment tools and correlated the results using AUC (Area Under the Curve) and Kaplan–Meyer survival plots.

### Testing for Batch Effects in TCGA Data

High throughput data generation are prone to batch effects as a result of variations in the equipment and/or reagent kits utilized at different sites or the skill level of the handling personnel, and other factors. TCGA samples were prepared and processed in batches at various locations of the consortium; therefore, the data could be subject to batch effect vulnerability. However, our Mbatch analysis has not identified any batch effects in the DNA methylation (Supplementary Figure S2a), gene expression (Supplementary Figure S2b), or miRNA expression data (Supplementary Figure S2c).

### Pre-filtering of Liver-Specific Gene Expression Data

A total of 386 liver-specific genes were removed from our analysis because the expression of these genes would likely result from the contamination of bile tissue with normal liver cells (Farshidfar et al., 2017). In addition, 80 other genes that showed at least five-fold elevated mRNA expression in the liver in comparison to all other tissues in The Human Protein Atlas (HPA) database (Uhlen et al., 2015) were also removed. In total, we removed the methylation and expression data of 466 liver-specific genes from further analysis.

### Unsupervised Analysis of Epigenome and Transcriptome Data

**FIGURE 1:**
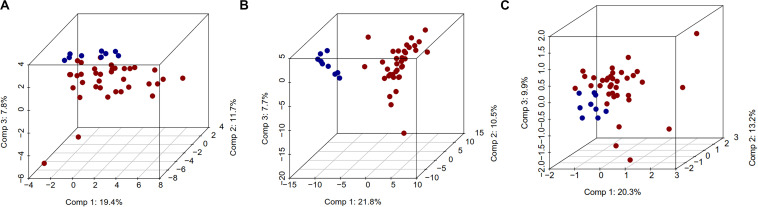
Three dimensional PCA plot for DNA methylation, gene expression, and miRNA expression data. In this plot, normal samples are in blue color and tumor samples in red color. For PCA analysis, we used complete DNA methylation, gene expression, and miRNA expression data. 3D PCA plots **(A)** DNA methylation, **(B)** gene expression, and **(C)** miRNA.

The unsupervised analysis of epigenome and transcriptome data using PCA showed excellent separation amongst normal and tumor samples. The first principal component (PC1) data alone had the ability to separate normal and CCA samples, with differences of 19.4, 21.8, and 20.3%, for DNA methylation, gene expression or miRNA expression respectively (Figure 1). Similarly, Pearson’s correlation matrix- and Manhattan distance matrix-based heatmaps showed distinctive patterns in tumor and in normal samples (Supplementary Figures S3a,b), which indicates that these individual data types by themselves can provide the discriminating information between normal and CCA samples.

### Genome-Wide Analysis of CpG Sites

**FIGURE 2:**
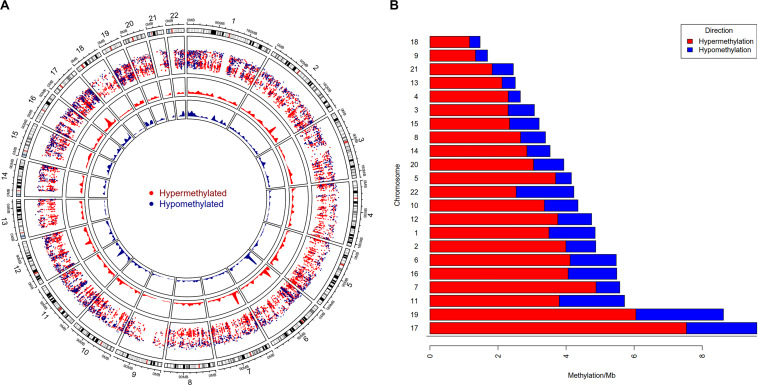
Genome-wide differential DNA methylation pattern in Cholangiocarcinoma. **(A)** Distribution of differentially methylated CpGs on each chromosome. Chromosomes are shown in a clockwise direction from 1 to 22 in the outermost circle; sex chromosomes (X or Y) were omitted from the analysis. The two innermost circles display differential hypermethylation and hypomethylation frequencies in a 10 Mb sliding window throughout the genome. The penultimate outer circle with red and blue dots correspond to hypermethylated and hypomethylated CpGs, respectively, where the distance of each dot (GpG) from the inner line represents log10 of the base-pair distance between two nearest CpGs. **(B)** Differential DNA methylation frequency for each chromosome (per Mb) in CCA. We assessed hypermethylation and hypomethylation frequency per Mb for each chromosome and sorted chromosomes based on their frequency.

To study the global DNA methylation patterns in CCA, we used the Illumina annotation file to remove all probes that are associated with liver-specific genes. A total of 12,259 differentially methylated CpGs (henceforth mentioned as dm-CpGs) were detected between tumor and normal samples; out of these 9,534 were hypermethylated and 2,725 were hypomethylated (Supplementary Figures S4a,b and Supplementary Table S1). Even at greater thresholds (Δβ ≥ 0.3), the number of dm-CpGs only drops to 9,443 showing a strong pattern of differential methylation across the genome. Figure 2A shows the global distribution of all the dm-CpGs on 22 human chromosomes. Chromosome 1 carried the maximum number of dm-CpGs, while chromosome 18 had the lowest. The two inner circles represent the density of hyper and hypomethylation in a 10 Mb sliding window throughout the genome. Distribution of dm-CpGs in ten different sub-regions of the genome is shown in Table 1 and Supplementary Figures S5a,b. List of top ten hypermethylated and hypomethylated CpG sites with corresponding gene, AUC, *p*-value log2 fold change and chromosome associations are available in Table 2.

**TABLE 1:** Total number of differentially methylated CpG sites in different genomic sub-regions.

Subregions	Δβ ≥ 0.1	Δβ ≥ 0.2	Δβ ≥ 0.3
3′UTR	723	463	351
5′UTR	1769	1052	823
1st Exon	1046	563	463
Gene Body	6988	4451	3319
TSS200	1565	799	627
TSS1500	2516	1405	1091
Island	7081	3643	2855
Shore	5981	3457	2631
Shelf	1556	1072	825
Open sea	6270	4087	3132

**TABLE 2:** Top twenty differentially hypermethylated and hypomethylated CpG sites in CCA.

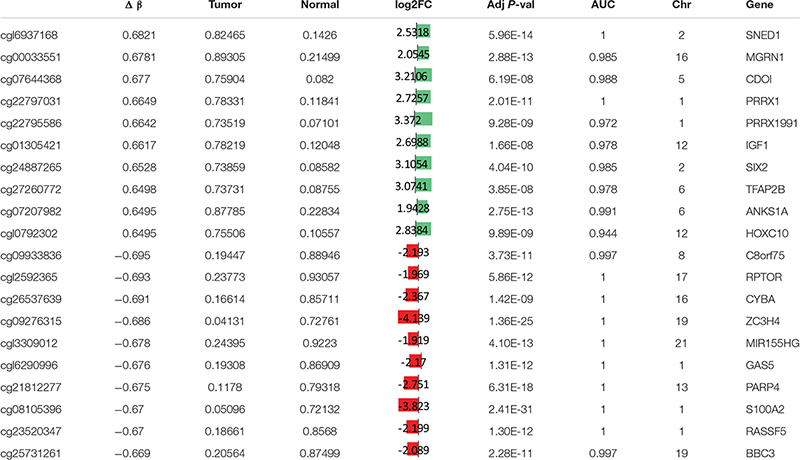

*Green bar represent hypermethylation and red hypomethylation.*

Our results demonstrated that chromosome 17 had the highest differential methylation frequency (9.61 dm-CpGs/Mb), while chromosome 18 had the lowest (1.47 dm-CpG/Mb) (Figure 2B and Supplementary Table S1). Methylation frequency of differential CpGs were calculated within a 10 MB sliding window to identify genomic regions with high-level epigenomic perturbations. Our analysis revealed that chr7:26500001-28000000 had the highest dm-CpGs frequency, this region was mostly hypermethylated. But, there is a sub-region in this region which is hypomethylated, this sub-region contains several overexpressed genes, including HOXA3, HOXA9, HOXA10, HIABDH, LOC441204 and hsa-miR196b, with the exception of the HIABDH segment. The genomic region on chromosome 6 (30000001 – 31000000) has the highest hypomethylation frequency. This genomic region has four overexpressed genes (DDR1, NRM, RNF39, and SFTA2), and no underexpressed genes.

In the current analysis, we also performed differential methylation analysis for CpGs which are within 10Kb up- or downstream from TSS of lncRNA or within the intron/exon of lncRNA (Zhi et al., 2014). A total of 484 CpG sites (unique 435 CpGs) associated with 265 lncRNAs were differentially methylated (Supplementary Table S1); of these, 387 are hypermethylated and 97 are hypomethylated. Details of methylation in the sub-regions of lncRNAs are provided in Supplementary Table S1.

### Genome-Wide Analysis of Differentially Methylated Regions (DMRs)

**FIGURE 3:**
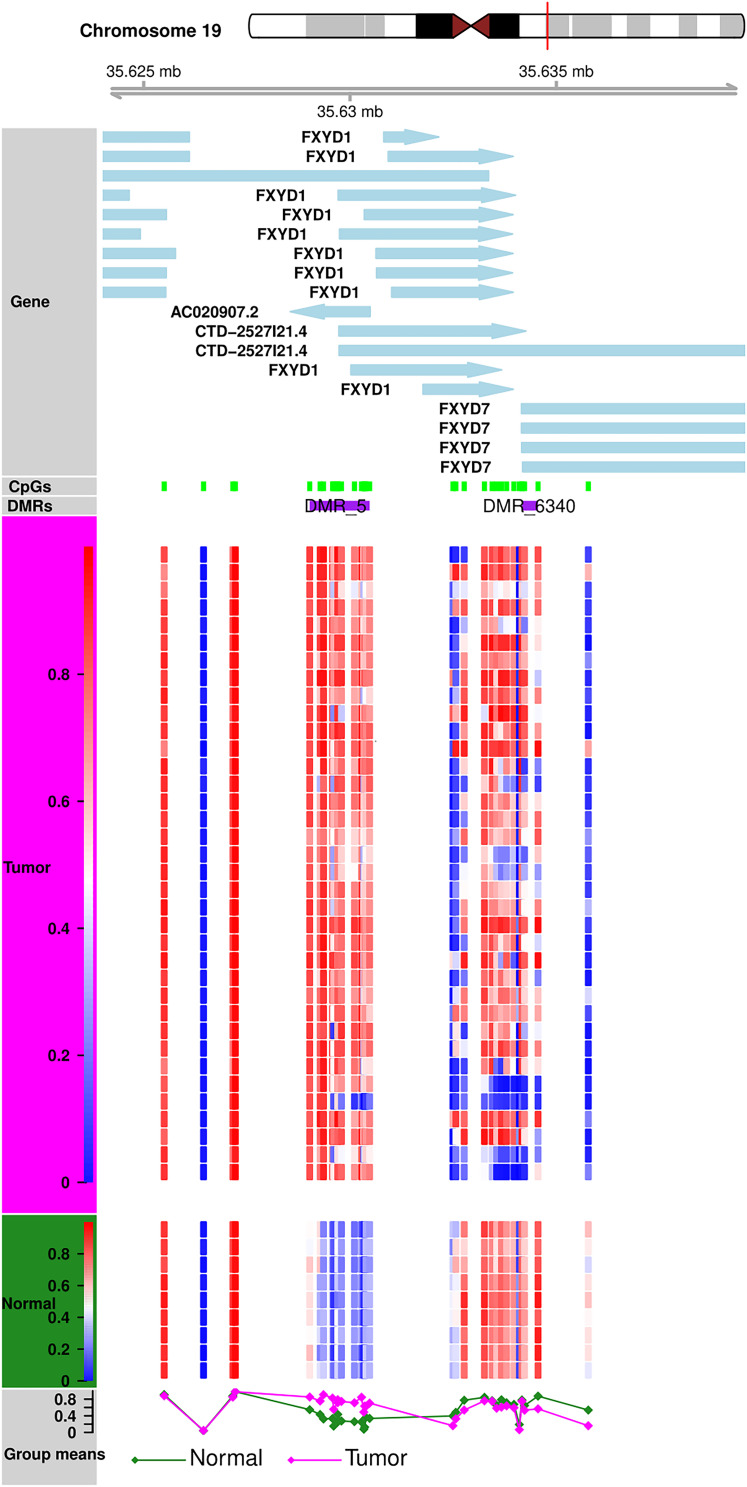
DMR Plot for one of the DMR. DNA methylation patterns in tumor and normal samples. Tumor and normal samples have distinct DNA methylation patterns for all CpGs in DMR.

Methylation patterns of different sub-regions of genes may have diverse consequences on gene expression. To conduct the DMR analyses, we removed all CpGs on the sex chromosomes to remove gender bias. Only those DMRs with two or more CpGs and at least one of them with a BH-adjusted *p*-value < 0.01 were considered as significant. We identified a total of 6,419 DMRs across the whole genome; Chromosome 1 showed the highest (610) and Chromosome 21 showed the fewest (Hu et al., 2016) of DMRs (Supplementary Table S2). Of the 6,419, we excluded 24 short DMRs (<10 bps) from further analysis. We observed characteristic DNA methylation patterns all across the genome that distinguished the tumors from the normal samples. Examples of DMRs showing contradictory methylation patterns between normal and tumor samples on chromosome 19 (Figure 3) and chromosome 2 (Supplementary Figure S6) are displayed.

### Analysis of DMRs in the Regulatory Regions

We mapped 6,395 DMRs against 65,933 known super-enhancer regions (Khan and Zhang, 2016) and observed that about half of them (Khan et al., 2012) have at least 10% length overlap with 16,233 super-enhancers. Next, we mapped these 3,109 DMRs against 1,867,665 DNase hypersensitive site clusters (Consortium, 2012) and found that about 97% of them also overlapped with 5,432 DNase hypersensitive site clusters. Out of these, 59 DMRs also exhibited at least a 10% length overlap when mapped against 2,311 Vista enhancers (Visel et al., 2007) (Supplementary Table S2). Thus, our findings suggest that the DMRs discovered in this study have significant roles in the overall transcriptional regulation of corresponding genes in cholangiocarcinoma.

### Differential Gene Expression Analysis

We observed 3,453 and 3,401 differentially expressed genes (DEGs) from *DESeq2* and *edgeR* analyses, respectively, with 3,305 DEGs intersecting between the two sets which were forwarded for further analysis. The logistic regression analysis identified 96% of these DEGs with an ROC-AUC value of ≥0.70, which means that a normal tumor classification can be achieved by using any single gene expression value at 70% accuracy. Out of these, 2,250 and 914 DEGs were upregulated and downregulated, respectively (Supplementary Figure S7a and Supplementary Table S3).

### Differential miRNA Expression Analysis

We found 101 differentially expressed miRNAs (DE-miRNAs), of which 58 were upregulated and 43 downregulated (Supplementary Figure S7b and Supplementary Table S3). Logistic regression analysis suggested that 98% of the DE-miRNAs also have an AUC of at least 0.75. Six miRNAs, i.e., miR-139, miR-187, miR-483, miR-598, miR-625, and miR-675 that were previously reported as bile duct cancer biomarkers (Wang M. et al., 2015) were also identified in our study. Apart from these, miR-21, miR-92b, miR-125a, miR-135b, miR-141, miR-196a, miR-200a, miR-200b, miR-200c, and miR-439 were upregulated, while miR-122, miR-148a, miR-152, miR-378c, miR-383, miR-483, miR-675, and miR-855 were downregulated (Supplementary Table S3). All of these miRs were earlier reported as differentially regulated in cholangiocarcinoma (Meng et al., 2006; Chen et al., 2009; Kawahigashi et al., 2009; Braconi et al., 2010; Karakatsanis et al., 2013; Wang S. et al., 2015; Zhang et al., 2015).

### Clustering Analysis

**FIGURE 4:**
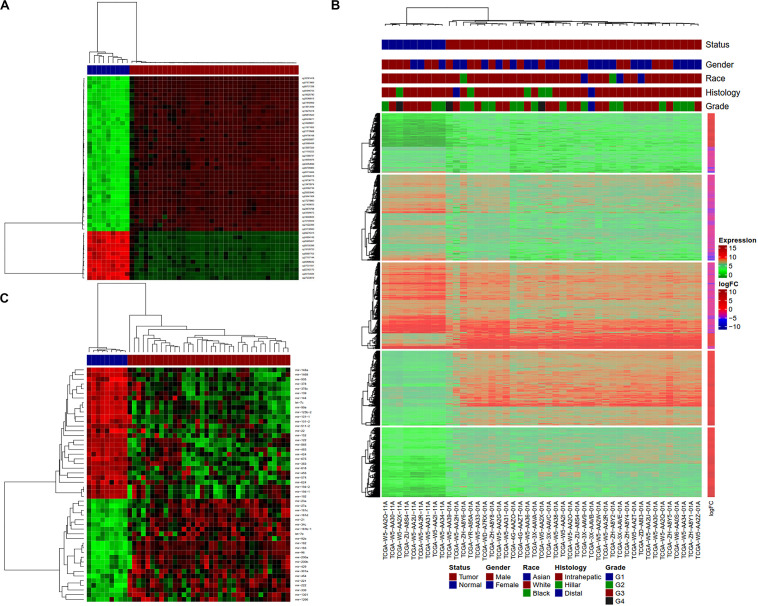
Clustering analysis of normal and tumor CCA samples using Dm-CpGs and DEGs. **(A)** Heatmap plot based on the top 50 differentially methylated CpG sites. **(B)** The vertical sidebar shows expression fold change in tumor Upper horizontal bars represent sample annotations of on disease status, gender, race, histology and tumor grade. **(C)** Heatmap plot based on the top 50 differentially expressed miRNAs.

Hierarchical clustering analyses were performed with *hclust* function in R on tumors and normal samples using dm-CpG, DEG and DE-miRNA expression values. Clustering analysis showed that tumor and normal samples have very different gene/miRNA expression and DNA methylation patterns (Figures 4A–C). All the samples were clustered along with their disease status in the clustering analysis.

### Pathway Enrichment Analysis

**FIGURE 5:**
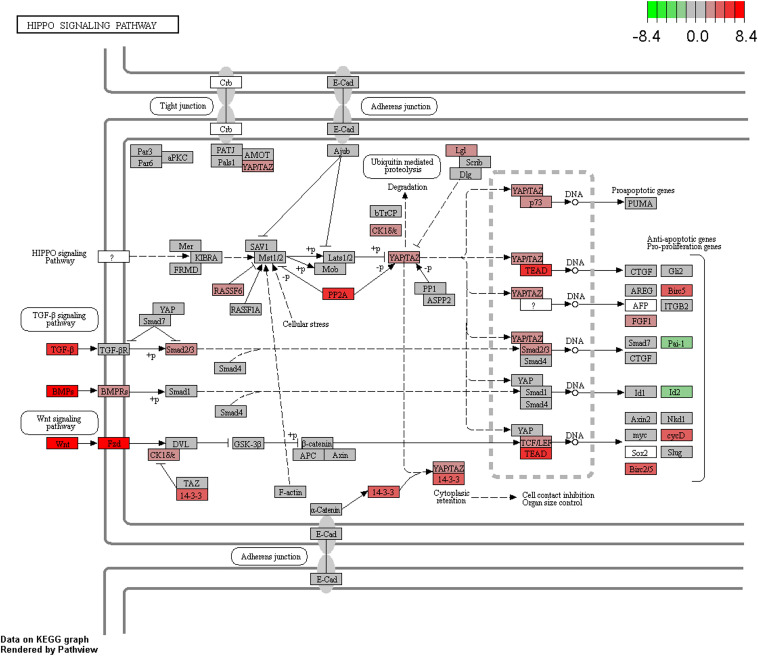
Enrichment of the Hippo signaling pathway in CCA. Upregulated/downregulated genes in the Hippo signaling pathway, color gradient shows the log2 fold change in tumor vs. normal analysis.

Pathway enrichment analysis of differentially expressed genes was carried out using *clusterProfiler* (Yu et al., 2012) and GSEA (Subramanian et al., 2005). We used a weighted score (-log10 of *DESeq2* FDR *p*-value multiplied with the sign of log2 fold change) for GSEA analysis (Davoli et al., 2017). KEGG, Biocarta, Reactome and PID pathways from MsigDB-v.5.2 (with the number of genes in the pathways ranging from 5 to 500) were used for further analyses. We identified that cell division, DNA replication, and hippo signaling pathways (Figure 5) were enriched with upregulated genes, while amino acid metabolism, glucose metabolism, drug metabolism, and autophagy pathway were enriched with downregulated genes (Supplementary Table S4 and Supplementary Figures S8a,b). Further, “*camera*” module in the *limma* tool was used for pathway enrichment analysis. DAVID and *camera* analyses also indicated the enrichment of nearly identical pathways as those indicated by GSEA and *clusterProfiler*.

Testing for over-representation of genes that are negatively correlated between miRNA expression and gene expression using *clusterProfiler* showed the enrichment of pathways such as cell division, bile secretion, amino acid metabolism, PPAR signaling, fatty acid degradation, etc. Enrichment analysis of genes whose expressions were negatively correlated with miRNA expression and additionally contained differentially methylated CpGs in their promoter region also indicated the same pathways. KEGG pathway analysis of genes with dm-CpGs pointed to the enrichment of pathways related to cancer, cell division and differentiation, amino acid metabolism, degradation of xenobiotics, and immune response (Supplementary Table S4). Hippo signaling pathway is also affected by DNA methylation (FDR corrected *p*-value = 3.70e^–18^).

### Correlation of DNA Methylation and Gene Expression

Pearson’s correlation was used to discover the relationship between DNA methylation and gene expression using R function, *cor.test*. Any correlation with a rho value of ≥0.25 and a BH adjusted *p*-value < 0.05 was considered significant. A significant correlation between methylated CpG sites within 1.5 kb from TSS and corresponding gene expression was observed in 5,252 CpGs, out of which 4,842 CpGs had a negative correlation and only 409 had a positive correlation (Supplementary Table S5).

Next, the correlation between locus-based DNA methylation for all dm-CpGs and the effect on gene expression within 100 kb on either side of those dm-CpGs was determined. This is to estimate the extent of multiple gene expression that is influenced by local DNA methylation in cholangiocarcinoma. Expression quantitative trait loci (eQTL) based linear regression (*eMap1* function) in an R tool, *eMap* (Sun, 2010) was used to estimate non-zero Pearson correlation between DNA methylation and gene expression for genes that have a TSS within 100 kb of a dm-CpG. The eQTL analysis enables us to ascertain the locus of the genome (eQTL) having variation in DNA methylation that impacts the expression levels of one or more genes given that locus (within 100 kb in either direction). Finally, we observed that a total of 3,097 dm-CpGs were significantly correlated with the expression of 1,401 unique genes in cholangiocarcinoma (Supplementary Table S5).

**FIGURE 6:**
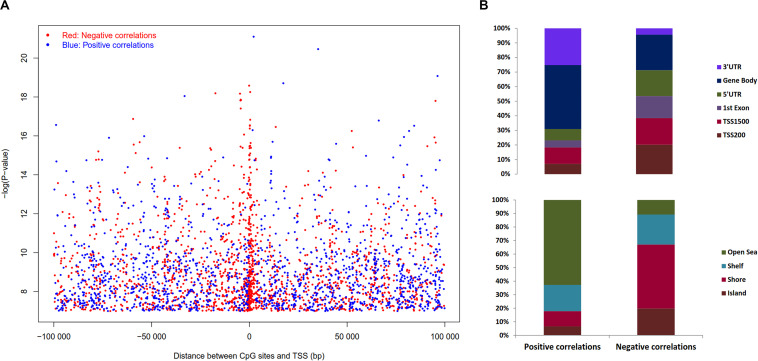
Distribution of the CpG sites whose DNA methylation levels were strongly associated with gene expression with BH adjusted *P*-value < 0.05. **(A)** Distribution of CpGs sites significantly correlated with corresponding gene expression. The negative logarithm of BH adjusted *P*-value of the correlation between DNA methylation β value and gene expression plotted against distance between CpG sites and transcription start site (TSS). Red dots denote negative correlation and blue dots denote positive correlation, sex chromosomes were excluded from the analysis. **(B)** Distribution of CpGs correlated with gene expression in sub-regions. Percentage stacked plot of the distribution of the negative and positive correlation corresponding to the functional regions of genes. Positive and negative correlations distribution patterns are quite different for the genomic regions.

The expression levels of 1,450 genes were positively associated with the DNA methylation levels, while 1,647 genes showed a negative correlation. Positively correlated CpG sites were quite evenly dispersed at both up and downstream of TSS (Figure 6A), while negatively correlated CpG sites were more abundant close to the TSS (2,000 bp up and downstream from TSS). The CpG sites associated with gene expression were dispersed throughout the entire genome, but chromosomes 1, 6p, 11q, 12, 16, 17, 22 are highly enriched (Supplementary Table S5).

Pearson’s correlation was assessed using R function, *cor.test* to estimate the association between DNA methylation in ‘gene sub-regions’ (3′ UTR, gene body, 5′ UTR, 1st exon, TSS200 and TSS1500) and the expression of corresponding genes. The expression of a total of 4,236 genes significantly correlated with DNA methylation levels in at least one subregion (Supplementary Table S5), where 3,633 genes were negatively correlated and 603 genes were positively correlated. Approximately 56% of the negative correlations were located in the upstream regulatory regions, i.e., promoter and 5′UTR, while ∼82% of the positive correlations were noticed outside of the promoter regions (TSS200 and TSS1500). In brief, upstream regulatory regions differential methylation lead to more negative correlations with gene expression as expected, and the opposite effect was observed in the case of the downstream methylation (Figure 6B). Similarly, CpG islands and its adjacent regions exhibited 89% of the negative correlations, while 63% of the positive correlations were away from islands (Figure 6B). In summary, methylation of CpG islands that are in close proximity to the TSS region was negatively associated with gene expression, while regions that are far away from islands were positively correlated. Similar trends were observed when we used Spearman’s correlations (Supplementary Figure S9).

### Correlation of Micro-RNA and mRNA Expression

We used differentially expressed miRNA and mRNA for correlation analysis. Associations between miRNA-mRNA were considered significant if Pearson’s correlation was ≥| 0.8| with a BH corrected *p*-value cut off at 0.05, by using the Bioconductor tool *MiRComb* (Vila-Casadesus et al., 2016). We observed 1,200 significant miRNA-mRNA associations, which contained unique sets of 585 genes and 31 miRNAs; as expected, all of them were negatively associated (Supplementary Figure S10 plot for one correlation example). Out of these 585 genes, 146 genes were differentially methylated with Δβ ≥ 0.2 in the promoter region (within 1.5 kb from TSS). Because these genes are differentially regulated at multiple levels, we refer to these 146 genes as genes under multiple regulatory control (GMRCs).

We observed 55 GMRCs in our analysis that were both miRNA-regulated (negatively correlated with gene expression) and promoter region methylation-regulated (negatively correlated with gene expression). We hypothesize that these genes could play vital roles in cholangiocarcinoma. This is further described below by our network analysis. When we implemented the over-representation test for these genes using *clusterProfiler*, we observed the enrichment of bile secretion, complement and coagulation cascades and ABC transporter pathways with a BH adjusted *p*-value < 0.025 (Supplementary Table S5).

### Network Analysis for GMRCs

Based on available interactome information, a total of 55 GMRCs were used to examine the regulatory network. The top five sub-networks were selected on the basis of the average ranking (AR) score from the network of GMRCs. To identify the molecular processes affected by these sub-networks, we performed Fisher’s exact test based overrepresentation analysis of canonical pathways using IPA software program. Among the cell cycle-related pathways, estrogen receptor (ER) signaling pathway is the most enriched. IGF-1 signaling pathway in signaling, acute phase response signaling in the inflammatory response, aryl hydrocarbon receptor signaling in development, coagulation system in immune response, and leucine degradation in amino acid metabolism stood out as the top enriched pathways (Table 3). However, enrichment of the hippo signaling pathway was observed in all the five sub-networks. The IPA canonical pathway analysis revealed that these GMRC genes were associated with critical cellular functions associated to cell cycle, growth and proliferation, cancer, amino acid metabolism, sulfonation, inflammation, and immune response (Supplementary Table S4).

**TABLE 3:** IPA canonical pathway analysis for the GMRCs and its first neighbors.

Ingenuity Canonical Pathways	−log(*p*-value)	Ratio	GMRC
Acute Phase Response Signaling	13.2	0.206	EP300
Estrogen Receptor Signaling	13.2	0.312	POLR2A
Glucocorticoid Receptor Signaling	12.8	0.222	POLR2A
Molecular Mechanisms of Cancer	11.3	0.136	EP300
Aryl Hydrocarbon Receptor Signaling	11.1	0.207	EP300
RAR Activation	11	0.179	EP300
Prolactin Signaling	9.95	0.253	EP300
p53 Signaling	9.73	0.216	EP300
IGF-1 Signaling	8.86	0.226	CTCF
LPS-stimulated MAPK Signaling	8.71	0.23	EP300
Adipogenesis pathway	8.65	0.254	POLR2A
PPAR Signaling	7.5	0.274	POLR2A
FGF Signaling	7.35	0.222	CTCF
TGF-β Signaling	7.05	0.276	POLR2A
EIF2 Signaling	7	0.195	POLR2A

*Here we used –log of FDR *p*-value in the table.*

### Effect of Transcription Factor (TF) Binding Motif Methylation on Gene Expression

Coherence between hyper and hypomethylated CpG sites in the known TF-binding motifs was investigated to identify the master regulator transcription factors in CCA. The Bioconductor tool, *ELMER* was used to identify differentially methylated distal enhancers (10 kb away up or down from TSS) and promoter (within 2 kb up or down from TSS) region probes in the TF-binding motifs and their effect on gene expression in CCA. For this, we used the JASPAR and Factorbook human TF binding motif database.

#### Effect of Enhancer Region Methylation on Gene Expression

We used both *p*-value and BH adjusted *p*-value cutoff of 0.01 for differential methylation analysis of distal enhancer region, and observed 16,801 and 19,577 probes significantly hypermethylated and hypomethylated, respectively in CCA. In the case of the hypermethylated, 8,043 distal enhancer probes demonstrated a statistically significant correlation with gene expression (Supplementary Table S6). A total of 91 Factorbook and JASPAR TF binding sequence motifs contained a minimum of 10 hypermethylated probes each. Finally, we associated the average β value of all distal enhancer probes within ± 100 bp of an enriched motif with the 1,982 known human TF genes’ expression (Ravasi et al., 2010). Finally, we observed a total of 29 TF binding motifs whose DNA methylation is significantly associated with TF gene expression (Supplementary Table S6). All TFs that fall within the top 5% of known motif-TF pair ranking are considered potential upstream regulators of TF genes. Similarly, in the case of hypomethylation, 12,251 probe-gene pairs showed a statistically significant correlation (Supplementary Table S6) and are also enriched in 91 Factorbook and JASPAR TF binding sequence motifs with minimum 10 probes per motif. Similarly, 31 TF binding motifs having a statistically relevant association with known TF genes’ expression were observed. Our analyses suggest that SOX7/10, FOXN3, FOXM1, RXRA, RORA, STAT3, TLX1, TEAD2, MAF, and HNF4A genes are the top potential upstream master regulators of distal enhancers (Supplementary Table S6).

#### Effect of Promoter Region Methylation on Gene Expression

Promoter regulatory analysis showed that a total of 10,120 hypermethylated probes within 2 Kb from TSS, had statistically significant correlations with corresponding nearest gene expression. A total of 91 Factorbook and JASPAR TF binding sequence motifs were enriched with a minimum 10 significantly hypermethylated probes for each TF. We found 20 TF binding motifs whose DNA methylation has significantly associated with the corresponding TF gene’s expression (Supplementary Table S6). For hypomethylation, a total of 6,489 probe-gene pairs showed a statistically significant correlation. These probes were enriched against 91 Factorbook and JASPAR TF binding sequence motifs with at least 10 statistically significant hypomethylated probes in each TF motif region. Finally, a total of 21 TF-binding motifs showed a strong association with known TF gene expression regulators. CREB3L3, ETS2, ET4, and TEAD2 genes are the top potential upstream regulators in CCA (Supplementary Table S6).

### Survival Analysis

Fisher’s exact test and univariate Cox-regression survival analysis suggest that age, race, gender, tumor stage, tumor grade, neoplasm stage, and family history of cancer have no role in the overall survival of CCA patients (Supplementary Figure S11). Our survival analysis of promoter methylation reveals that a total of 10 differentially hypermethylated and 5 hypomethylated CpGs are linked to CCA patients’ overall survival (Supplementary Table S7). In the case of gene expression, we observed that a total of 72 DEGs and two DE-miRNAs are also associated with CCA patients’ survival (Supplementary Table S7). We found that high and low expression groups have a significant difference in the overall survival with *p*-value < 0.05. Even at a rigorous p-value cutoff of 0.01, we observed twelve DEG, one DE-miRNA, and two hypermethylated CpG sites associated with survival, while none of the hypomethylated CpGs qualified by this criterion. Overall, analysis of the survival-associated genes, miRNA and promoter methylations resulted in the identification of potential prognostic biomarkers of CCA.

### Real-Time qPCR Based Genes Expression Validation in CCA Patients

Based on our bioinformatics analysis (Supplementary Table S7), we expanded our studies to validate the gene expression of four gene targets, MDK, HNF1B, PACS1, and GLUD1. All of these genes showed expression patterns similar to those observed in our bioinformatics analyses. There were significant differences in the expression of all four genes in CCAs as compared to adjacent normal (*p*-value 0.0009, 0.015, 0.001, and 0.03, respectively) (Figure 10). These results suggest that the expression of these genes would be useful in evaluating the risk for CCA.

## Discussion

Alterations in DNA methylation and miRNA expression are commonplace in a variety of tumors and these changes have been perceived as causative factors of oncogenesis in several cancer types (Merlo et al., 1995; Chu et al., 2016; Ramachandran et al., 2016). Such alterations significantly affect gene expression and this information has been effectively used to identify biomarkers that could discriminate the cancerous cells from normal cells (deVos et al., 2009; Oh et al., 2013; Li et al., 2014; Wang M. et al., 2015; Chu et al., 2016; Cheerla and Gevaert, 2017). In this study, we have conducted an exhaustive analysis of global DNA methylation, mRNA and miRNA gene expression, and made correlations among all data types followed by network, pathway and survival analyses. To our knowledge, this study is the first comprehensive and integrative analysis of CCA data from TCGA.

As shown in Figure 1, the DNA methylation and mRNA/miRNA expression patterns observed in the normal versus tumor samples are sufficient to discriminate the two classes in PCA analysis (Figure 1). This suggests that the classification of the tumor and normal samples using multiple molecular data profiles could help identify accurate and reliable biomarkers in CCA. The global methylation pattern of CCA suggests that dm-CpGs were dispersed across the genome. We found that Engrailed Homeobox 1 (EN1) gene had the highest number of dm-CpGs (Kramer et al., 2014), where most of them were hypermethylated in the gene body and none in the promoter region. Engrailed 1 (EN1), is exclusively overexpressed in extremely aggressive tumors (Beltran et al., 2014), we did observe overexpression of EN1 gene in our data. This is consistent with earlier reports that gene body methylation is positively associated with expression (Yang X. et al., 2014). Due to the extensive methylation of gene body CpGs, EN1 could be a key player in CCA.

Genomic sub-region that harbors HOXA3/A9/A10, HIABDH, LOC441204, and miR196b is hypomethylated, and showed overexpression of all of these genes in CCA except HIABDH (*p*-value > 0.05, corroborating the fact that DNA hypomethylation in general positively affects corresponding gene expression. DMRs are distributed across the genome (Supplementary Table S2), and they overlapped with known super-enhancer regions (Khan and Zhang, 2016) and ENCODE DNase hypersensitive site clusters (Consortium, 2012). Hence, these overlapping regions of DMRs (with the DNase hypersensitive, VISTA and super-enhancer regions) could serve as transcriptional hotspots in CCA.

Differential expression analysis of transcriptome data using several orthogonal tools (*DESeq2*, *edgeR* or *limma*) showed a very consistent list of genes. Pathway analysis suggested the enrichment of several important cancer-related pathways. Clustering analysis showed separate clusters for the tumor and normal samples (Figure 4B). Overall, our results demonstrate that CCA tissues display a unique gene expression profile compared to their adjacent normal tissues.

Previous CCA gene expression analyses suggest the overexpression of S100A6, platelet-derived growth factor-alpha (PDGFA), neutral proliferation differentiation and control protein 1 (NPDC1), while the cytochrome P450, succinate dehydrogenase (SDHA), isocitrate dehydrogenase 2 (IDH2) and glutathione S-transferase-alpha4 (GSTA4) was downregulated (Wu et al., 2011). We also observed similar expression trends for these genes (Supplementary Table S3). Similarly, genes that encode proteins associated with cell growth and metastasis, e.g., mucin 13 (MUC13), carcinoembryonic antigen-related cell adhesion molecule 5 (CEACAM5), FXDY3, epithelial cell adhesion molecule (EPCAM), transmembrane channel-like 5 (TMC5), and ets homolog factor (ETH) are overexpressed, which corroborate previous reports in CCA (Subrungruanga et al., 2013). Cell division, DNA replication related pathways, Hippo signaling pathway were highly represented with upregulated genes, while amino acid metabolism, glucose metabolism, drug metabolism, autophagy pathways were represented by downregulated genes. Similar trends were observed in CCA (Wu et al., 2011), and more specifically, on the importance of Hippo signaling (Rizvi et al., 2016) (Figure 5) and amino acid metabolic (Murakami et al., 2015) pathways in CCA were reported. Overall, our results suggest that differential gene expression impacts cell division/differentiation, amino acid metabolism and autophagy pathways in cholangiocarcinoma (Supplementary Table S4).

From our analysis of miRNA expression data, we observed a number of differentially expressed miRNAs that include six previously identified potential biomarkers of CCA (Wang M. et al., 2015. The pattern of expression of certain of these miRNAs matches with several known CCA related miRNA (Chen et al., 2009; Kawahigashi et al., 2009; Karakatsanis et al., 2013; Wang S. et al., 2015; Zhang et al., 2015). It’s been known that miR-21 and miR-200b, both oncogenes (Meng et al., 2006; Wang L. J. et al., 2015), are overexpressed in CCA, while miR-122 (Wang L. J. et al., 2015), which is a tumor suppressor is downregulated. Another tumor suppressor, miR-1258, which is recently identified as a prognostic biomarker for several other cancers (Zhang et al., 2011; Hu et al., 2016; Shi et al., 2017) is also downregulated in CCA. Classification AUC of 1 of miR-122 and miR-1258 suggest their use as potential diagnostic biomarkers for CCA. Other known miRNAs, miR-148a and miR-152, which target DNA methyltransferases 1 (DNMT1) are also downregulated in our analysis corroborating similar reports (Braconi et al., 2010). We also observed the overexpression of miR-196b in CCA and a similar trend was observed in gastric (Shao et al., 2018) and head and neck cancers (Alvarez-Teijeiro et al., 2017). Overall, the differential expression profiles of miRNAs in CCA were distinct enough to separate normal and tumor samples using clustering analysis solely based on the miRNA expression data (Figure 4C).

Correlation among gene expression and DNA methylation of CpG sites within 100 kb of TSS showed that negatively correlated CpG sites (hypermethylated CpGs which lowered gene expression) are primarily located adjacent to TSS in the upstream region, while positively correlated ones are located in the downstream region to the TSS (Figure 6A). This result substantiates the key regulatory role of promoter DNA methylation on gene expression compared to non-promoter regions. Regarding different gene sub-regions, around 56% of the negative correlations were identified in the promoter regions (i.e., TSS200, TSS1500, and 5′UTR), and around 61% of the positive correlations were found in the non-promoter regions (Figure 6B). This supports that hypermethylation at the upstream regions of the gene predominantly results in a vital alternative biological phenomenon for gene silencing. Similar observations were also found in other cancers, including pancreatic (Mishra and Guda, 2017; Mishra et al., 2019), chronic lymphocytic leukemia (Kulis et al., 2012) and breast cancers (Fleischer et al., 2014).

The previous reports also showed that overexpression of several TFs, including FOXC1, was associated with CCA carcinogenesis (Li et al., 2013). FOXC1 is also an important biomarker of cancer which plays a central role in cell proliferation, cell differentiation, cell migration, survival and death and metastasis (Ray et al., 2010; Han et al., 2017). We also observed hypomethylation of FOXC1 TF binding motif and overexpression of FOXC1 in our study, which may have a key role in CCA. TEAD2 and TEAD4 are members of the TEAD family of transcription factors, both of which target oncogene, YAP1, which is an important protein of Hippo signaling (Diepenbruck et al., 2014; Tang et al., 2018). TEAD4 is already a known biomarker for breast cancer, colorectal cancer and prostate cancer (Zhou et al., 2016); its TF binding motif regions are hypomethylated in CCA.

Pathway analysis of the GMRC genes revealed that their functions are associated with bile secretion, ABC transporters, and complement and coagulation cascades. IPA canonical pathway analysis of the top 5 subnetworks of GMRCs and their first interactive neighbor observed several pathways related to cancer such as cell cycle and cell division, immune and inflammatory response, amino acid metabolism, etc. (Supplementary Table S4). These results suggest that GMRCs play a very important role in CCA progression.

**FIGURE 7:**
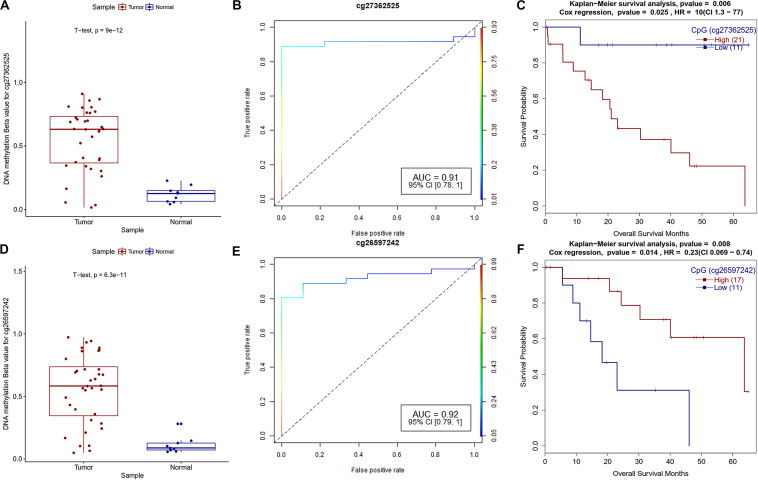
Survival plots of differentially methylated CpGs in CCA patients. **(A)** Boxplot for cg27362525 tumor and normal samples with *t*-test *p*-value. **(B)** ROC plot for cg27362525 for logistic regression classifier model. **(C)** Survival plot for high vs. low methylation group for cg27362525. **(D)** Boxplot for cg26597242 tumor and normal samples with *t*-test *p*-value. **(E)** ROC plot for cg27362525 for logistic regression classifier model. **(F)** Survival plot for high vs. low methylation group for cg27362525.

Survival analysis of promoter methylation identified ten hypermethylated and five hypomethylated CpGs that are strongly associated with the survival of CCA patients. The majority of them are either in CpG-islands or close to CpG-island (shore) (Supplementary Table S7). Survival associated hypermethylated CpGs are mapped mostly against the HOX family (HOXA9, HOXA10, and HOXB8), GABR1, and GALNT13 genes, while CpGs in CDK18, DDR1, and SFN genes were hypomethylated. GRBRA1 encodes a gamma-aminobutyric acid (GABA) receptor and promoter hypermethylation of this gene was already reported as a biomarker in colorectal cancer (Lee et al., 2012). As shown in Figure 7, hypermethylated CpG sites in the promoter and TSS regions of GABRA1 and UPS6 (Ubiquitin Specific Peptidase 6), respectively, showed very high AUC values, hence could serve as strong biomarkers of CCA. The effect of promoter methylation of the HOX family of genes in patients’ survival has not been reported previously, but promoter hypermethylation of HOXA9 in non-smokers is associated with recurrence-free survival (RFS) in non-small cell lung cancer (Hwang et al., 2015). Our analysis reveals the role of HOXA10 promoter hypermethylation in CCA patients’ survival. Recently, Shao et al. reported the critical role of HOXA10 promoter methylation in gastric cancer (Shao et al., 2018). We also observed hypermethylation of miR-196b and hypomethylation of lncRNA MIR100HG promoter CpG, which are both associated with CCA patients’ survival. It needs to be tested how promoter methylation of HOX family genes and other genes affect CCA patient survival.

Further survival analysis of DEGs identified several potential biomarker genes for CCA including MLL11, MDK, DEPDC1, SLC35E4, PLXDC1, PACS1, PIWIL4, GLUD1, all have AUC ∼1 with very low *p*-value (Supplementary Table S6). Real-time qPCR analysis also validated the significantly altered expression of MDK, PACS1, and GLUD1 in CCA (Figure 10), which supports their potential use as CCA biomarkers. Phosphofurin acidic cluster sorting protein 1 (PACS1) is a tumor-suppressor that regulates intrinsic (mitochondrial) apoptosis with its partner ADA3. In a recent study, Brasacchio et al. (2018) reported that patients with lowered expression of PACS1 had significantly low overall survival in gastric cancer. In our analysis, we also observed low expression of PACS1 is associated with low survival in CCA. Similarly, Fucosyltransferase IV (FUT4), associated with the proliferation and metastasis, is proposed as an effective biomarker for breast cancer diagnosis (Yan et al., 2015). We also observed overexpression of FUT4 in CCA (AUC = 0.996); however, patients with lower expression showed lower overall survival (*p*-value 8.7e-04).

**FIGURE 8:**
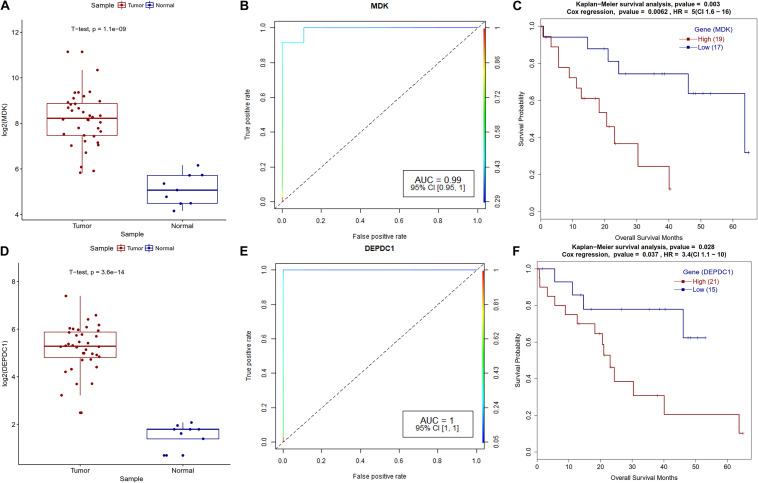
Survival plot for differentially expressed genes that correlate with CCA patients’ survival. **(A)** MDK gene expression boxplot for tumor and normal samples with *t*-test *p*-value. **(B)** ROC plot for MDK gene for logistic regression classifier model. **(C)** Kaplan–Meier plot for MDK-high and MDK-low methylation patient cohorts. **(D)** DEPDC1 gene expression boxplot for tumor and normal samples with *t*-test *p*-value. **(E)** ROC plot for the DEPDC1 gene for the logistic regression classifier model. **(F)** Kaplan–Meier plot for DEPDC1-high and DEPDC1-low expressing patient cohorts.

Midkine (MDK) is a heparin-binding growth factor that is overexpressed in various types of human cancers, but its clinical significance is still unknown in CCA. We also observed the overexpression of the MDK genes in the real-time qPCR analysis of CCA patients. It’s been reported that low MDK expressing patient cohorts have better survival and a similar trend was observed in our analysis (Figure 8). PIWIL4 belongs to the Argonaute family of proteins, which are involved in the development of organisms and maintenance of germline stem cells and that they are ectopically expressed in multiple forms of cancer (Hock and Meister, 2008). Wang et al. (2016) reported overexpression of PIWIL4 in breast cancer with a lowered overall patient survival. We observed a similar trend for PIWIL4 expression in CCA. DEP domain containing 1 (DEPDC1) plays a crucial role in tumor growth and metastasis (Huang et al., 2017); a previous study found that the shRNA knockdown of DEPDC1 in glioma cell lines and in mice have better survival (Kikuchi et al., 2017). Patients with high DEPDC1 expression have low survival in CCA (Figure 8).

On the other hand, we observed that patients showing higher expression of ID2 have better survival in CCA, which was also observed in acute myeloid leukemia (AML) (Ghisi et al., 2016). Similarly, expression of MLLT3 and MLLT11 genes that are well studied in leukemia are also found to be strongly associated with the survival of CCA patients. Several reports from other cancers showed that patients with high GLUD1, ANXA4, and PLXDC1 expression have low overall survival, but our findings in CCA are contrasting. On similar lines, patients having high CD24 expression were shown to have low metastasis-free survival in extrahepatic CCA (Kim et al., 2013), but we observed an opposite trend in our findings (Supplementary Table S7).

**FIGURE 9:**
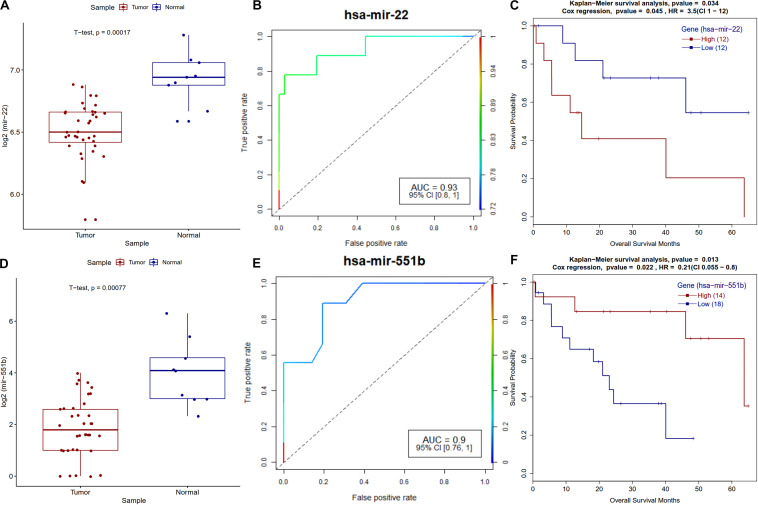
Survival plot of the survival associated differentially expressed miRNAs. **(A)** Boxplot for miR-22 expression in tumor and normal samples with *t*-test *p*-value. **(B)** ROC plot for miR-22 miRNA for the logistic regression classifier model. **(C)** Survival plot for high vs. low methylation group for miR-22 miRNA. **(D)** Boxplot for miR-551b expression in tumor and normal samples with *t*-test *p*-value. **(E)** ROC plot for miR-551b miRNA for the logistic regression classifier model. **(F)** Kaplan–Meier plot for miR-551b miRNA high vs. low expressing patient cohorts.

MicroRNAs are known to play a vital role in cancer progression, metastasis and survival of the patients. Survival analysis of differentially expressed miRNAs revealed that miR-22 and miR-551b have a strong correlation with the survival of CCA patients. A known tumor suppressor, miR-22 (Yang C. et al., 2014) is downregulated and its high expression is correlated with poor survival of patients in CCA (Figure 9), a similar trend was observed in other types of cancers (Zuo et al., 2015; Zou et al., 2017). Regarding miR-551b, it is downregulated in most of the CCA patients, high expression is associated with better prognosis and a similar trend was observed in gastric cancer (Song et al., 2017) (Figure 9).

**FIGURE 10:**
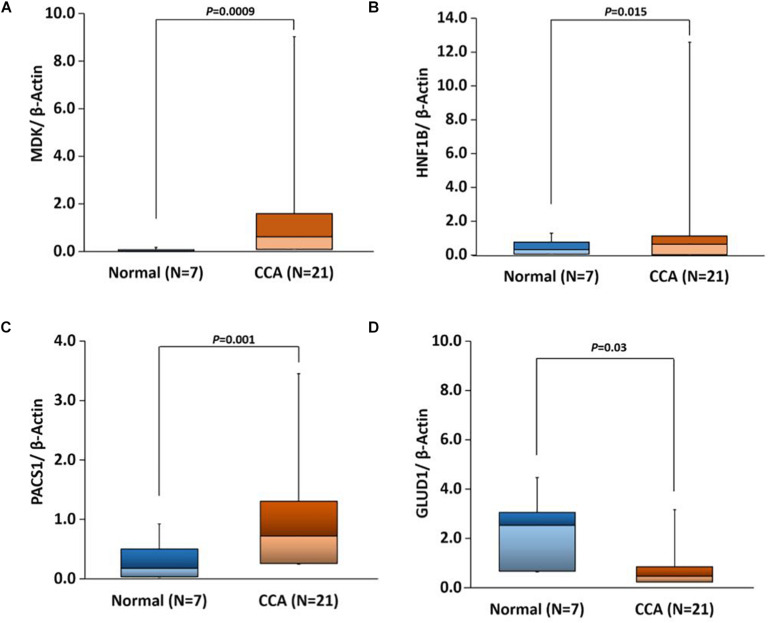
Box plots showing real-time quantitative PCR (qPCR) gene expression in CCA versus adjacent normal tissues with *t*-test *p*-values for the following genes. **(A)** MDK, **(B)** HNF1B, **(C)** PACS1, and **(D)** GLUD1.

## Conclusion

DNA methylation analysis of cholangiocarcinoma data shows significant changes in tumor methylomes compared to normal pancreatic tissues. To our understanding, this study represents the first genome-wide DNA methylation analysis of TCGA cholangiocarcinoma data. Clustering analyses based on methylation, gene or miRNA expression data show distinct clusters of the tumor and normal samples. Differential methylation of several known CCA biomarkers was observed in our study, confirming previous observations. DNA methylation region analysis reveals that several genomic regions have very high hypermethylation/hypomethylation frequencies, and expression of genes encoded from these genomic loci correlated accordingly. Pathway enrichment analysis of differentially methylated and differentially expressed genes shows that cell division, DNA replication-related pathways, and Hippo signaling pathway are the top upregulated pathways, while amino acid metabolism, glucose metabolism, drug metabolism, and autophagy pathways are the top downregulated. Our current analysis showed that Hippo signaling pathway was affected by promoter DNA methylation, gene expression, and GMRCs. This result has further confirmed the importance of hippo signaling in cholangiocarcinoma.

Differential expression of several known marker genes and miRNAs and differential methylation of promoter regions was observed in the current analysis. Real-time qPCR analysis further confirmed the change in gene expression of several genes in CCA. Most of the survival associated promoter CpGs are in the CpG-islands or proximal to CpG-islands. Survival associated promoter CpGs are mapped against genes that are either reported as a biomarker in other cancers or otherwise associated with cancers. High AUC and low survival p-value of these CpGs suggest that they can be further explored as potential biomarkers in CCA. Survival analysis of differentially expressed genes and miRNAs also identified several possible biomarkers for CCA, and a majority of these biomarkers are already identified in another type of cancers but none for CCA. Our analysis suggests that expression of DEPDC1, FUT4, MDK, PACS1, PIWIL4, miR-22, miR-551b, and DNA methylation of cg27362525 and cg26597242 could be explored further as potential biomarkers of cholangiocarcinoma.

## Data Availability Statement

Publicly available datasets were analyzed in this study. This data can be found here: https://portal.gdc.cancer.gov/projects/TCGA-CHOL.

## Author Contributions

NM and CG conceived and developed the study design. NM and SS performed the statistical analysis and produced the figures. MN did the DMR enrichment analysis. UM acquired samples from the University of Alabama at Birmingham (UAB) Tissue Biorepository and directed the experimental validation work. PB and AE performed real-time qPCR experiments. NM drafted the manuscript. CG edited and improved the manuscript. All the authors read and approved the final manuscript.

## Conflict of Interest

The authors declare that the research was conducted in the absence of any commercial or financial relationships that could be construed as a potential conflict of interest.

## Supplementary Material

Supplementary Table 1 Information of differentially methylated CpGs. Differential methylation information for lncRNAs and differential methylation frequency for each chromosome.

Supplementary Table 2 Information of differentially methylated regions (DMRs) in CCA. Information about DMRs overlaps with VISTA and super-enhancer and DNase hypersensitive regions.

Supplementary Table 3 Information about differentially expressed genes and miRNAs.

Supplementary Table 4 Results for pathway enrichment analysis for differentially expressed and differentially methylated genes. Table for the list of pathways for GMRCs and the top 5 subnetworks in GMRCs analysis.

Supplementary Table 5 Table for the correlations based on eQTL analysis and a correlation between genes and corresponding promoter CpGs. Table for the aggregate correlation of CpG in gene sub-region. Correlation between miRNA and gene expression.

Supplementary Table 6 Correlation between gene and corresponding hypomethylated/hypomethylated distal enhancer CpGs. Transcription factor (TF) motif analysis results for hypermethylation and hypomethylation for distal enhancers and promoters.

Supplementary Table 7 Survival analysis table for differentially expressed genes and miRNAs and, differentially methylated promoter CpGs.
