# Supplementary material for: Identification of Prognostic Markers in Cholangiocarcinoma Using Altered DNA Methylation and Gene Expression Profiles
Source: Front Genet. 2020 Oct 20;11:522125. doi: 10.3389/fgene.2020.522125 (PMC7606733; doi:10.3389/fgene.2020.522125)
Supplement: Supplementary Table 1 — Information of differentially methylated CpGs. Differential methylation information for lncRNAs and differential methylation frequency for each chromosome. [file Data_Sheet_1.docx]

Supplementary Material

# Supplementary Data

All supplementary data and tables are available online at <https://figshare.com/articles/TCGA_cholangiocarcinoma_analysis/11350037>.

# Supplementary Figures and Tables

## Supplementary Figures

**Supplementary Figure 1.** The figure legends are required to have the same font as the main text, 12 points normal Times New Roman, single-spaced. Please use a single paragraph for each legend and prepare the figures keeping in mind the PDF layout.


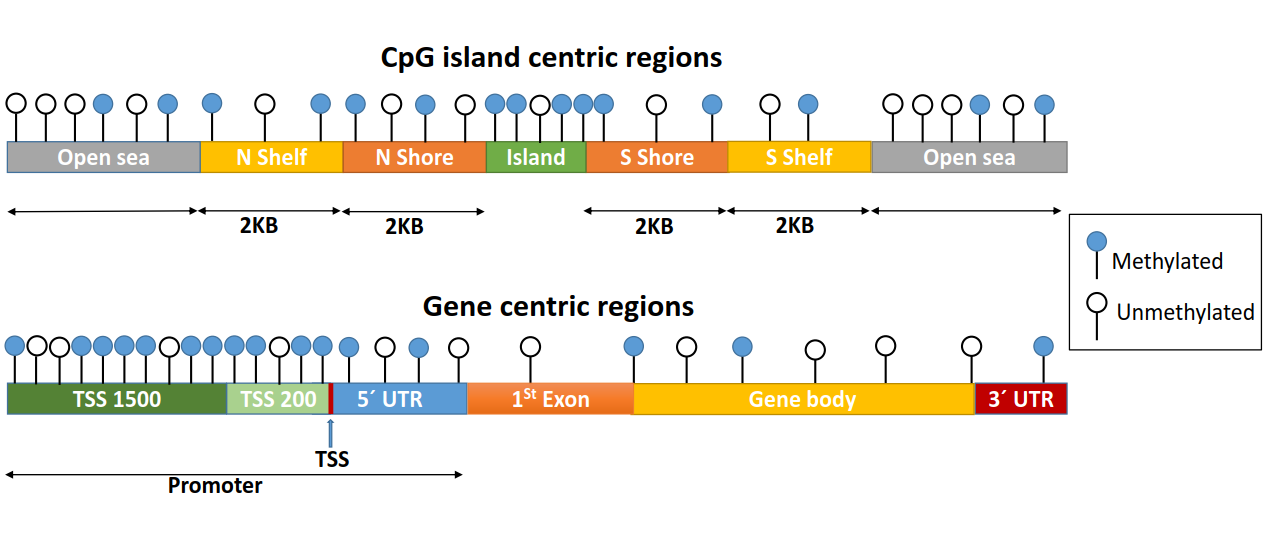


**Supplementary Figure S1: CpGs annotation based on CpG-islands and gene annotation.** CpG shores are defined as 2Kb upstream/downstream from the ends of the CpG islands, CpG shelves are defined as another 2Kb upstream/downstream of the farthest upstream/downstream limits of the CpG shores. The open sea is region that are away from the shelves.


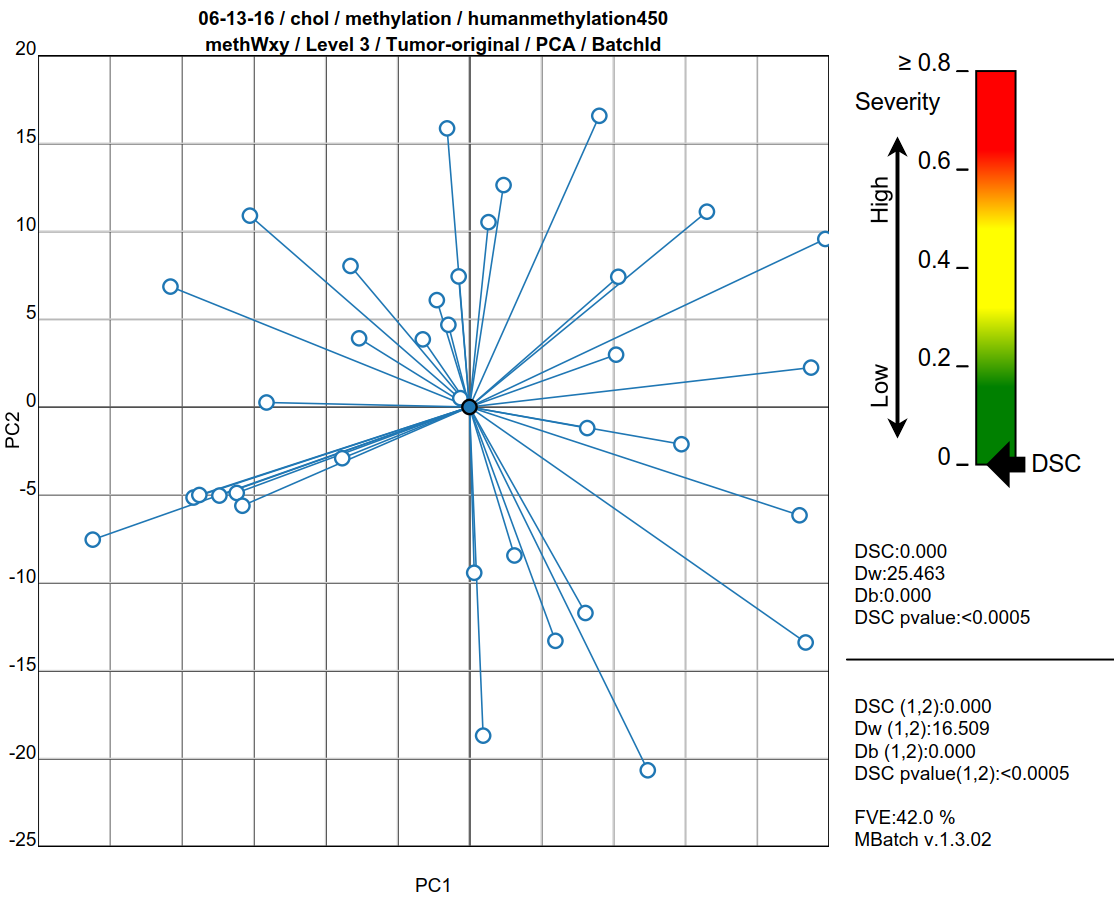


**Supplementary Figure S2.a: PCA plot of TCGA level3 DNA methylation data.** The plot suggests that there is no batch effect in level3 TCGA CCA DNA methylation data.


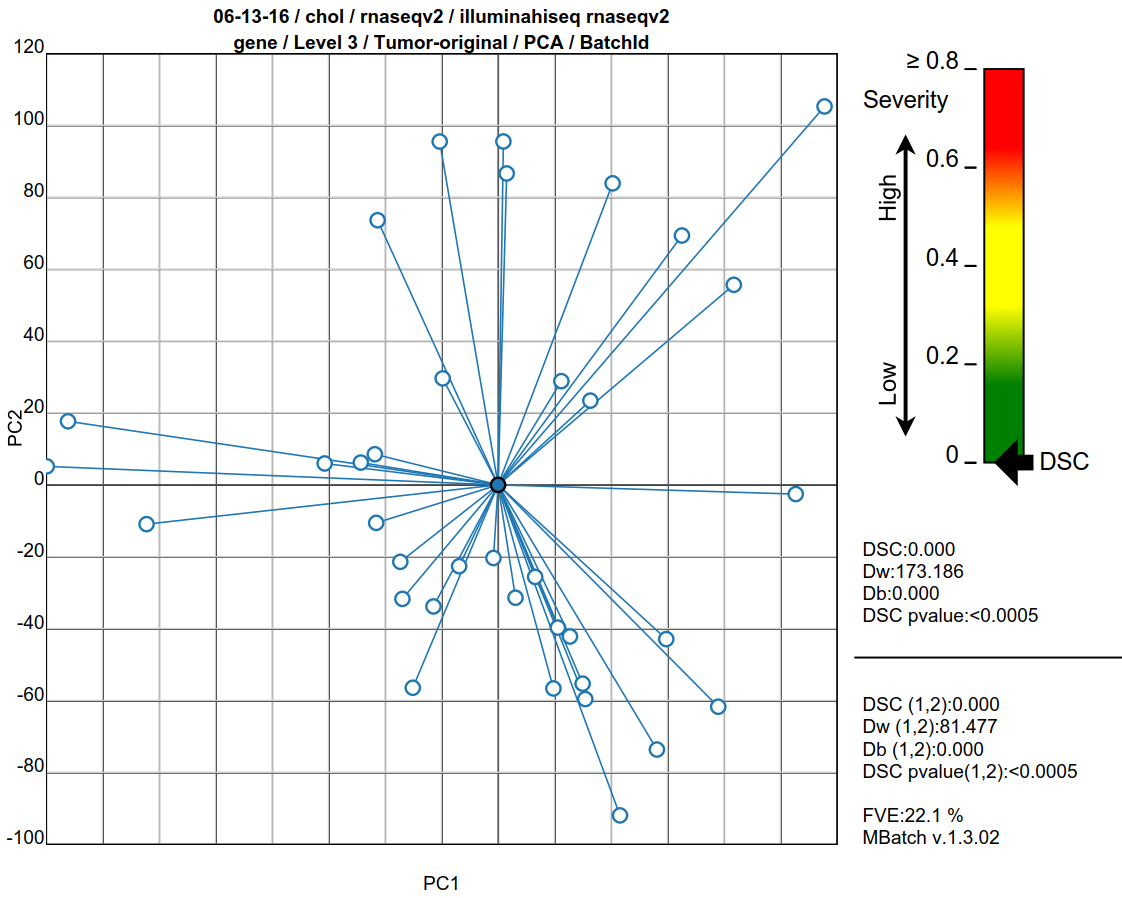


**Supplementary Figure S2.b: PCA plot of TCGA level3 gene-level expression data.** This plot shows that there is no batch effect in level3 RNASeqV2 gene expression data.


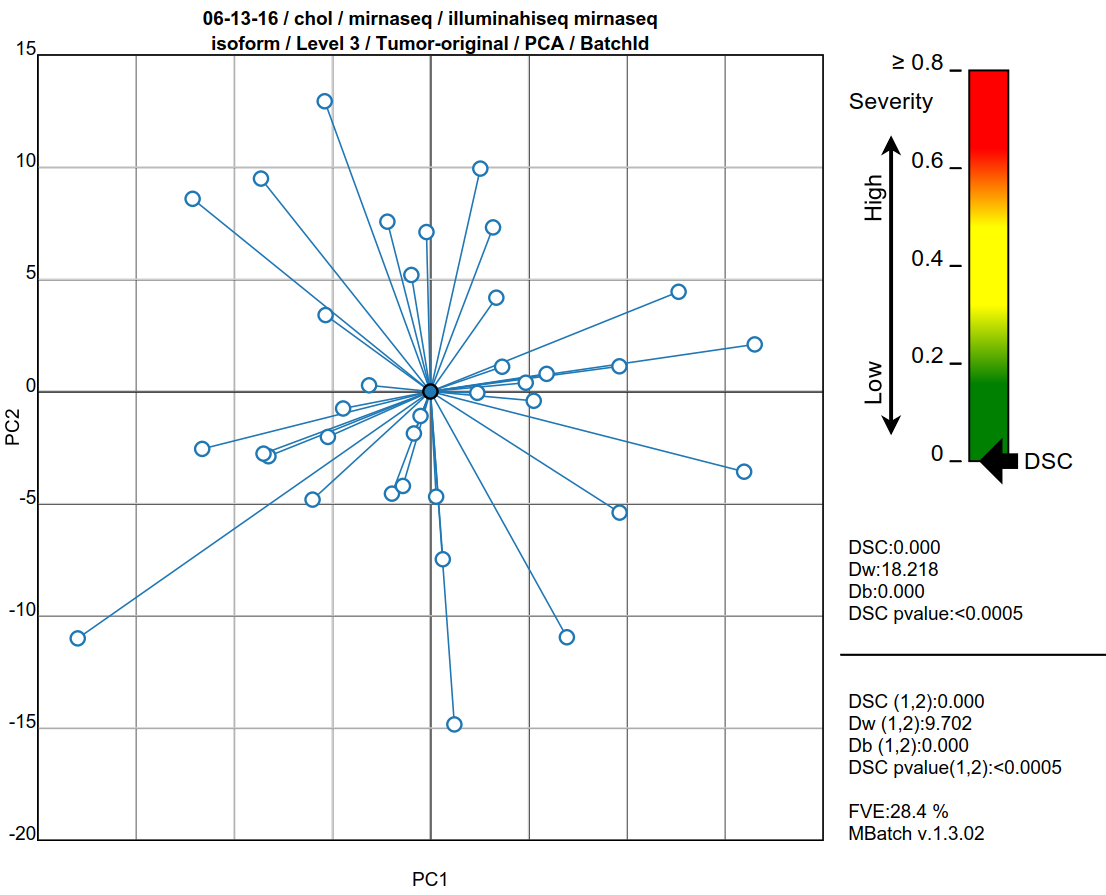


**Supplementary Figure S2.c: PCA plot of TCGA level3 miRNA expression data.** This plot shows that there is no batch effect in level3 miRNA expression data.


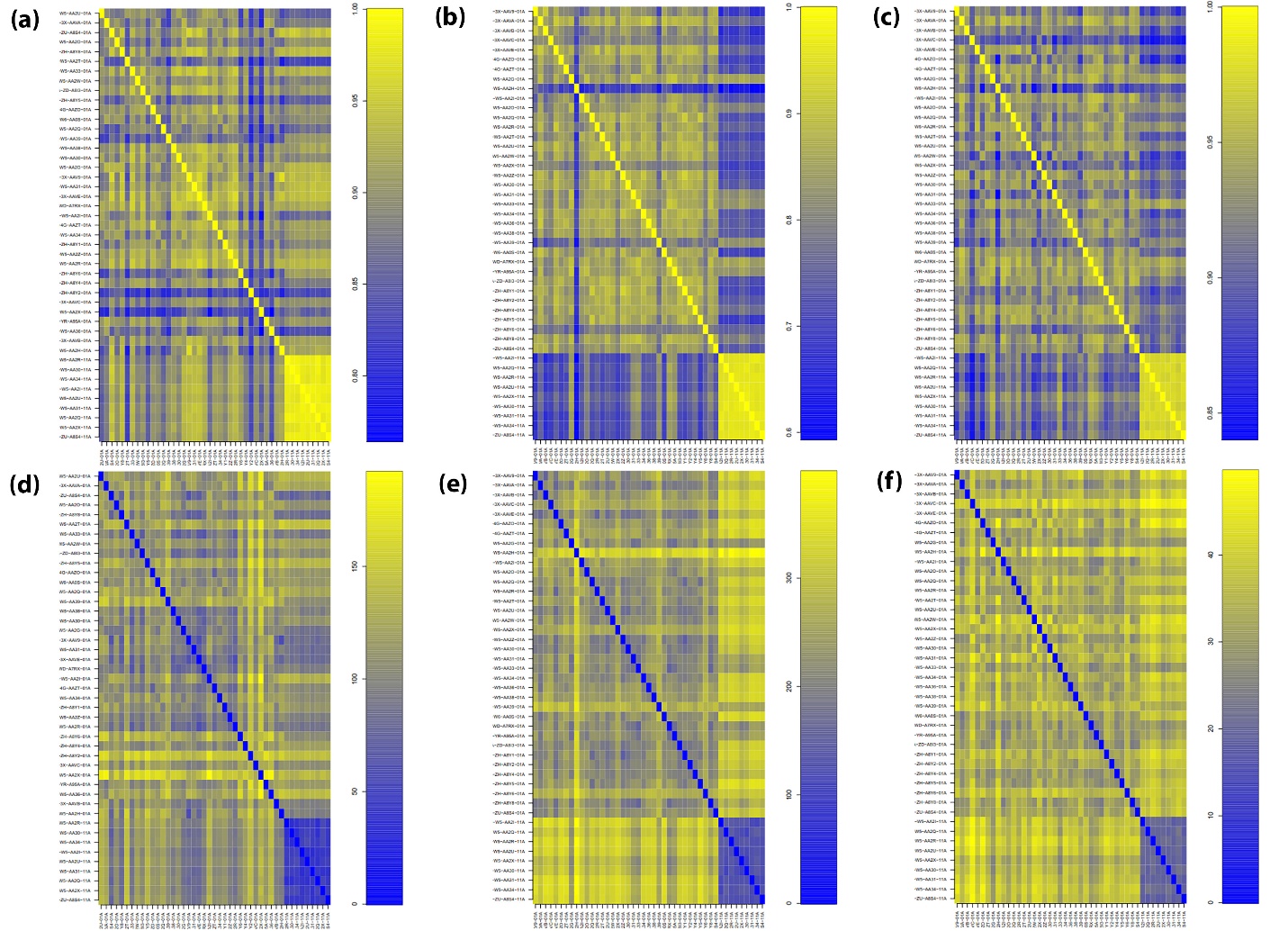


**Supplementary Figure S3: Correlation plot of TCGA level3 DNA methylation, gene-level expression, and miRNA expression.** In each plot, the top 36 are tumor samples and bottom 9 are normal samples. At the top (**Figure S3.a-c**) Pearson correlation plot for DNA methylation and mRNA and miRNA expression from left to right, bottom (**Figure S3.d-f**) Manhattan distance plot for DNA methylation and, mRNA and miRNA expression from left to right. For this plot we calculated Pearson correlation and Manhattan distance matrix by using all CpGs, genes and miRNA, the plot was generated in R.


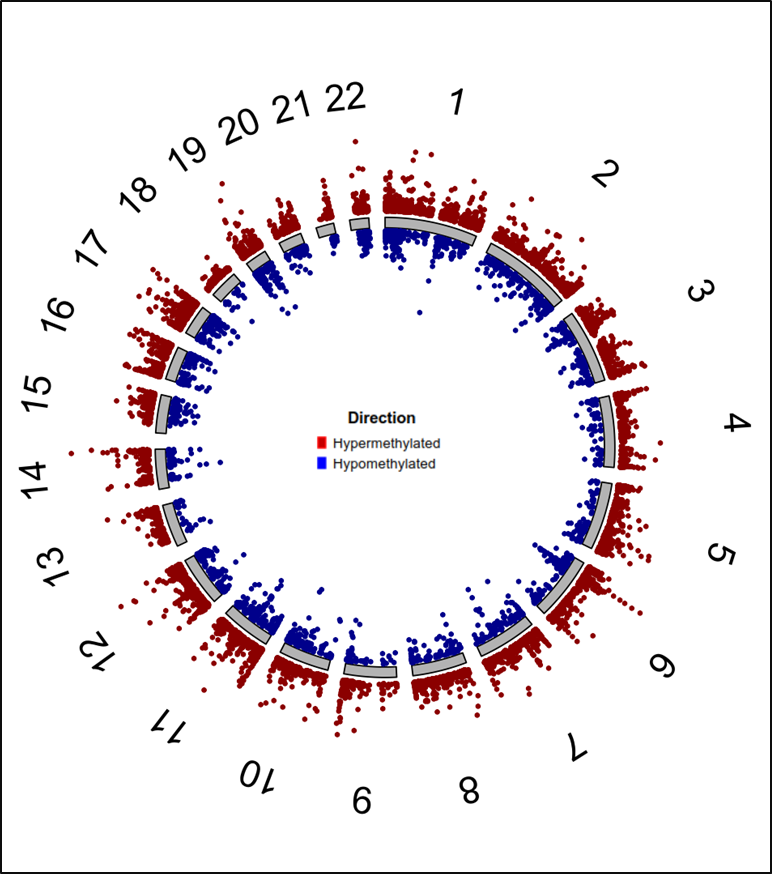


**Supplementary Figure S4.a:** Genome-wide differential DNA methylation patterns in CCA. The difference in DNA methylation in all CpG sites passing false discovery rate (FDR) with Δβ ≥ 0.1. Δβ is weighted by T-statistics (calculated by using *limma*) such that the distance from a central core (grey) indicates an increasing level of statistical significance. Chromosomes are shown clockwise from 1 to 22; we did not use sex chromosomes (X or Y) in our analysis.


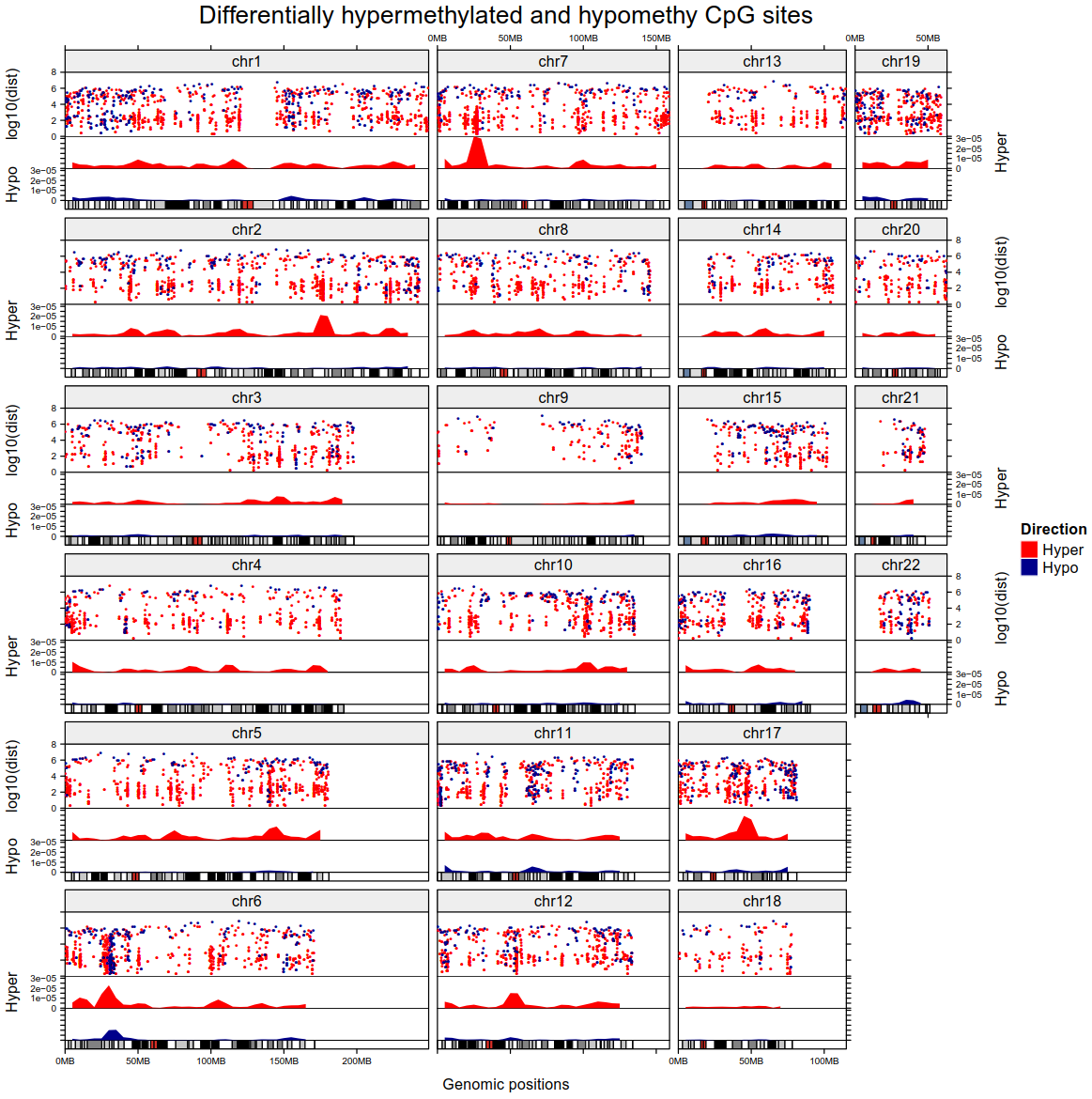


**Supplementary Figure S4.b: Distribution of differentially methylated CpGs**. The innermost line is an ideogram of chromosomes followed by the differential methylation frequency in 5 Mb sliding window across the genome. Blue and red dot for hypermethylated and hypomethylated CpGs in the outermost box, distance of each dots (GpGs) from the inner line is log10 of bp distance between two nearest CpGs.


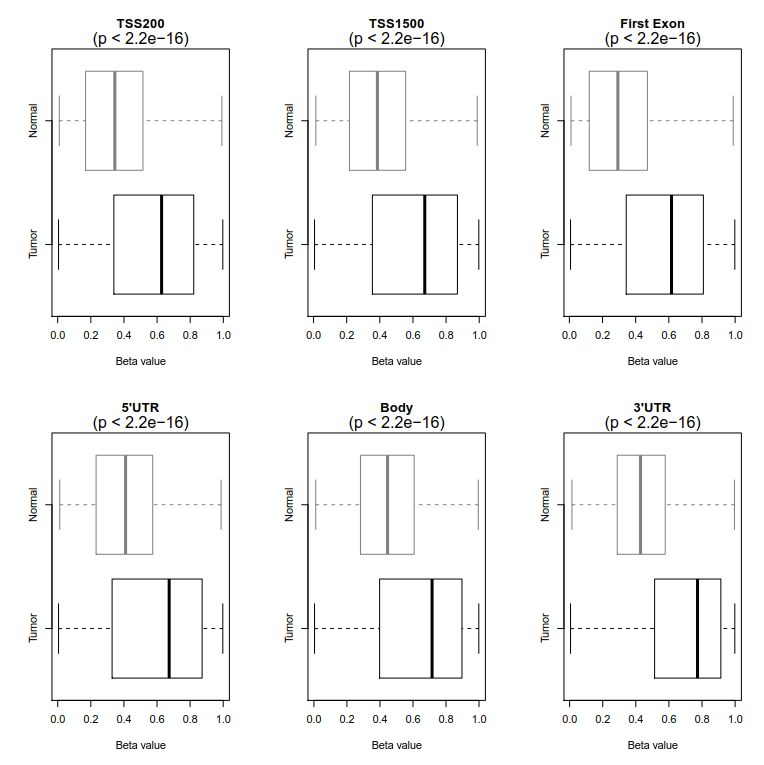


**Supplementary Figure S5.a: Boxplot using DNA methylation beta values in six different gene subregions.** Wilcoxon rank test was used to calculate the p-value in difference in DNA methylation in tumor and normal samples for each subregion.


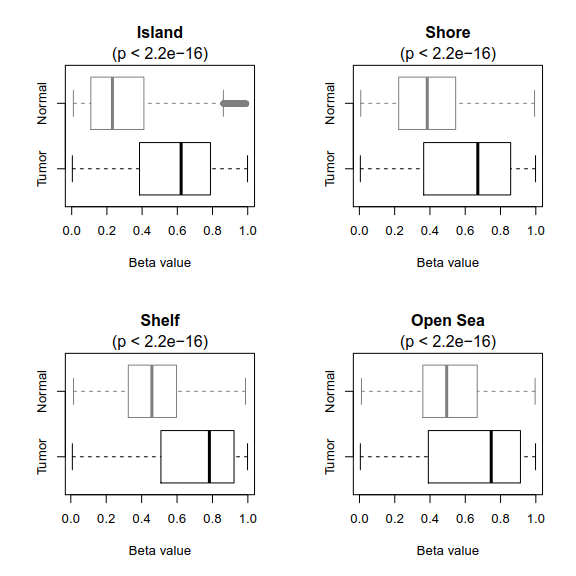


**Supplementary Figure S5.b: Boxplot for the DNA methylation beta values in four different UCSC-annotated genomic subregions.** Wilcoxon rank test was used to calculate the p-value indifference in DNA methylation in tumor and normal samples for each subregion.


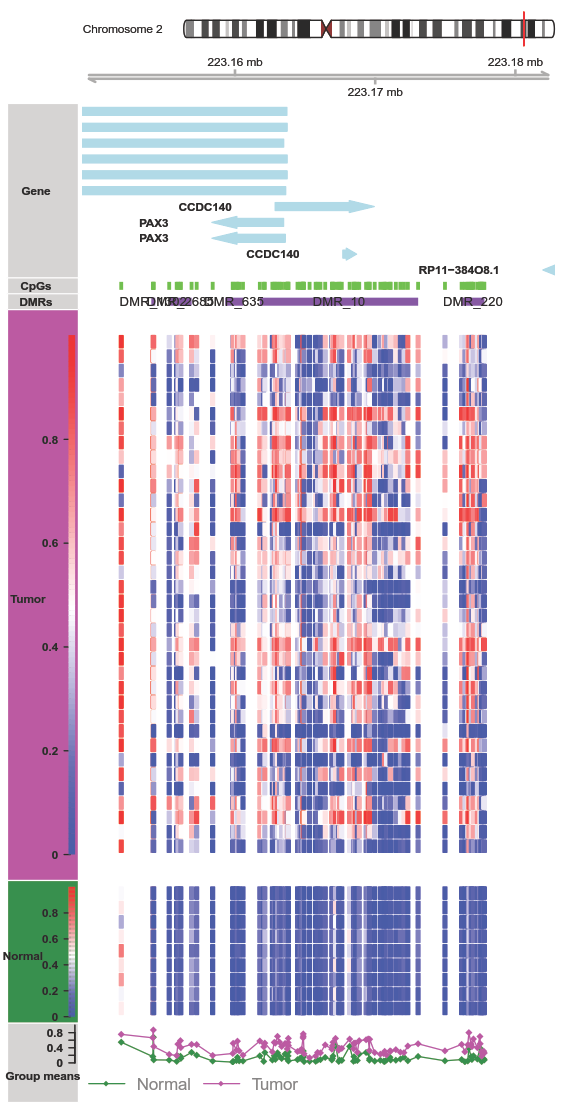


**Supplementary Figure S6: Plot illustrating a DMR on chromosome 2 and its DNA methylation patterns in tumor and normal samples**. Tumor and normal samples exhibit distinct DNA methylation patterns for all CpGs in these DMRs.


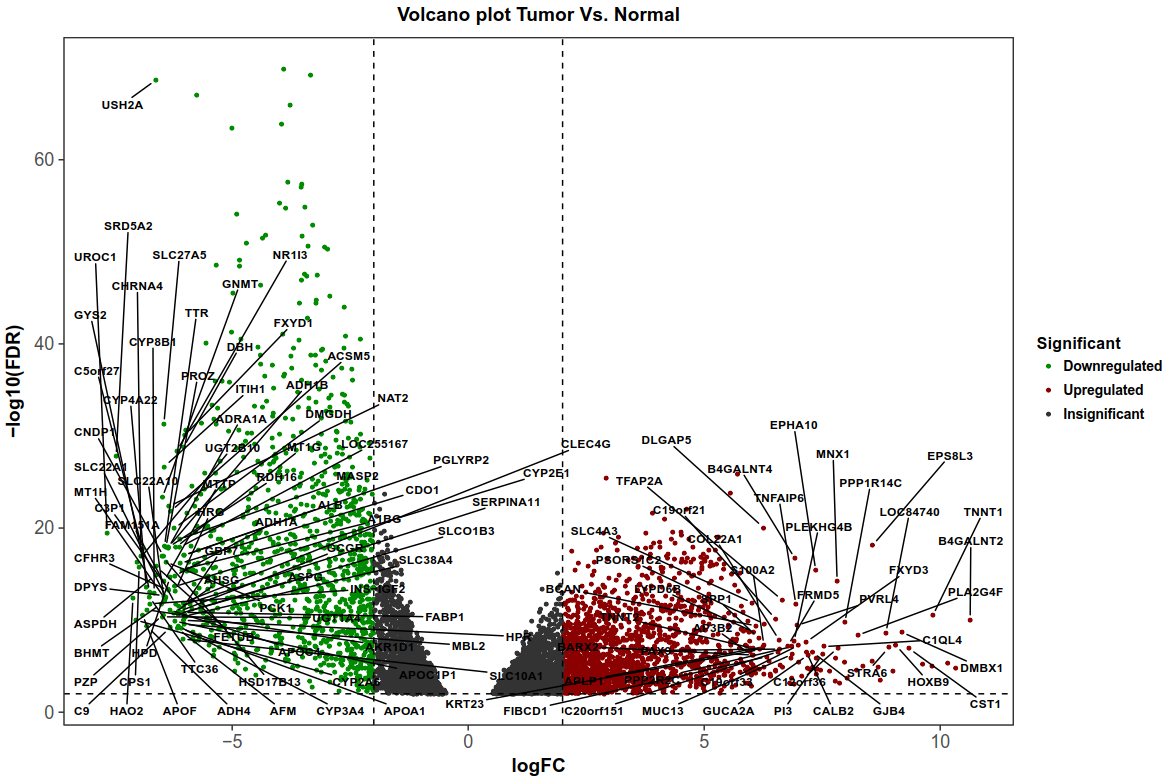


**Supplementary Figure S7.a: DEGs identified between normal and tumor samples of CCA.**  The x-axis shows the log2-fold change in gene expression between tumor and normal, and the y-axis shows the statistical significance of the differences (log_10_ (FDR)). Red dots represent overexpressed, and green dots represent underexpressed genes with FDR < 0.01, while the black dots represent genes whose expression levels did not reach statistical significance (FDR > 0.05). The names of only the most significant (logFC and log_10_ (FDR P-value) > 5) genes are displayed.


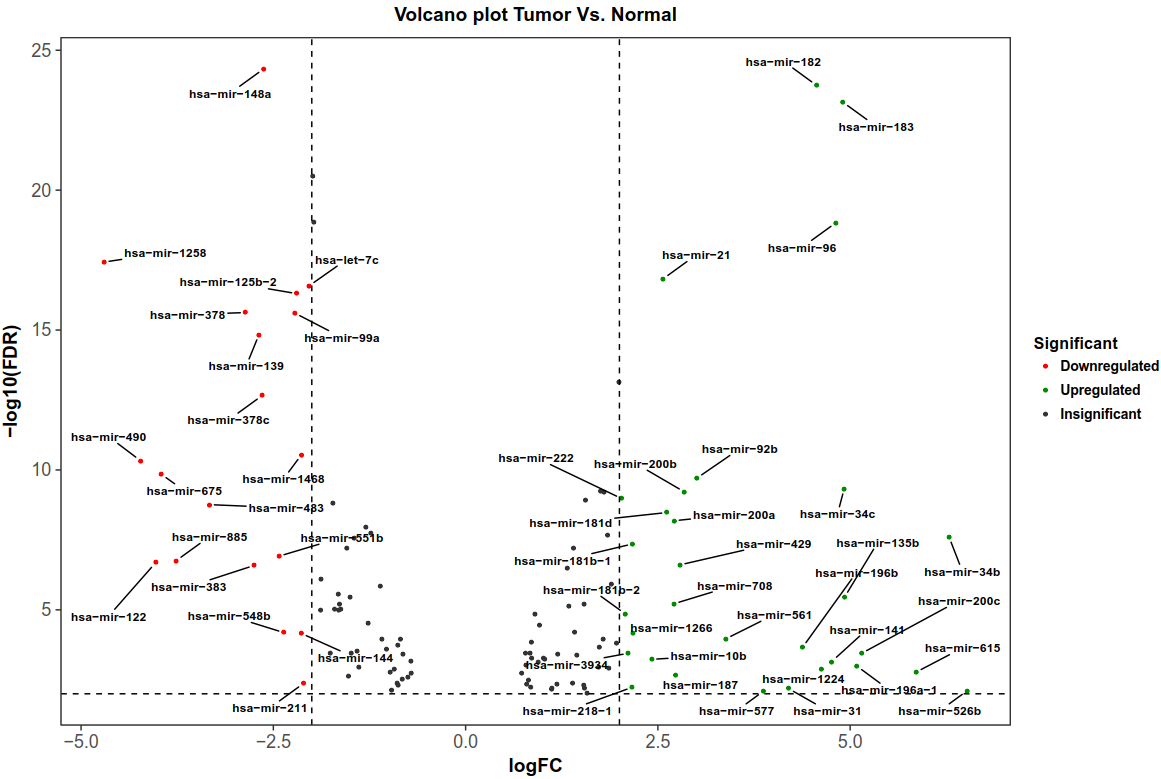


**Supplementary Figure S7.b: Differentially expressed miRNAs identified between normal and tumor samples of CCA.**  The x-axis shows the log2-fold change in gene expression between tumor and normal, and the y-axis shows the statistical significance of the differences (log_10_ (FDR)). Red dots represent overexpressed, and green dots represent underexpressed genes with FDR < 0.01; while the black dots represent genes whose expression levels did not reach statistical significance (FDR > 0.05). The names of only the most significant (logFC and log_10_ (FDR P-value) > 5) genes are displayed.


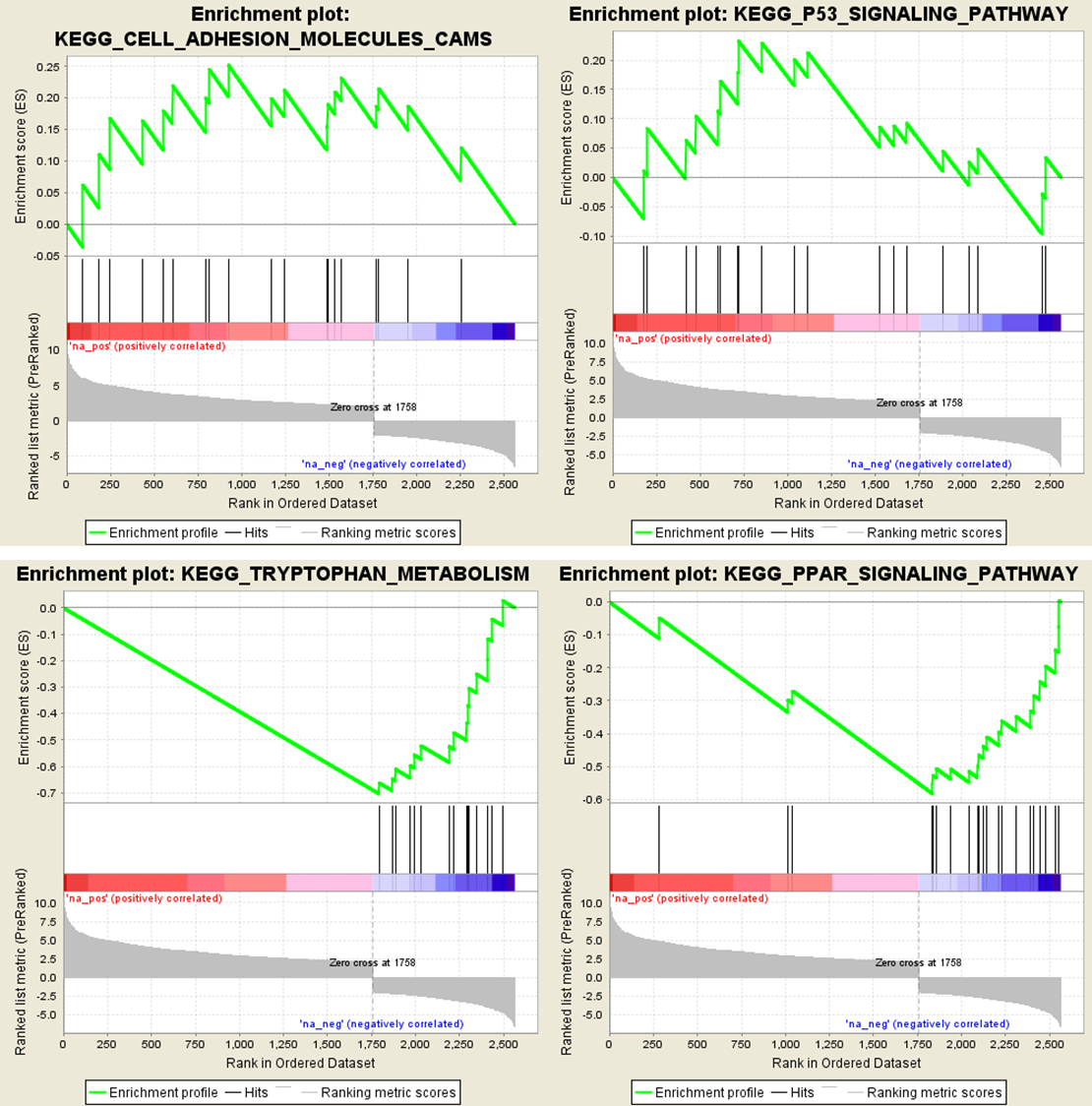


**Supplementary Figure S8.a:** Gene set enrichment analysis (GSEA) for the differentially expressed genes. For GSEA, we used KEGG canonical pathway and used FDR < 25% for significantly enriched pathways. The pathway enriched by upregulated in cancer at top and downregulated at the bottom.


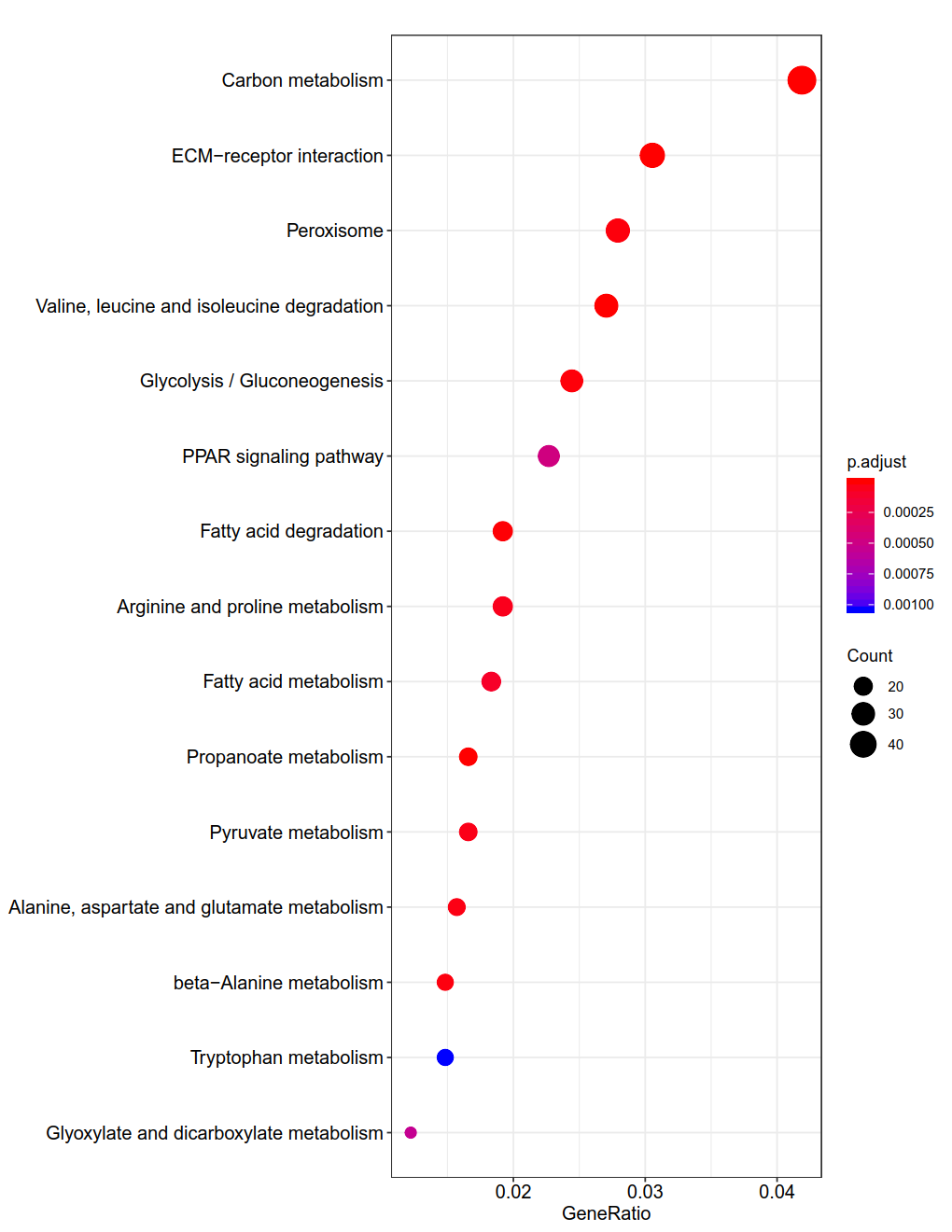


**Supplementary Figure S8.b:** GSEA for the differentially expressed genes by using KEGG canonical pathway in R/Bioconductor tool *clusterProfiler*. Significantly pathway enriched by DEG are plotted, size of the dot represents gene ration size and color for the FDR.


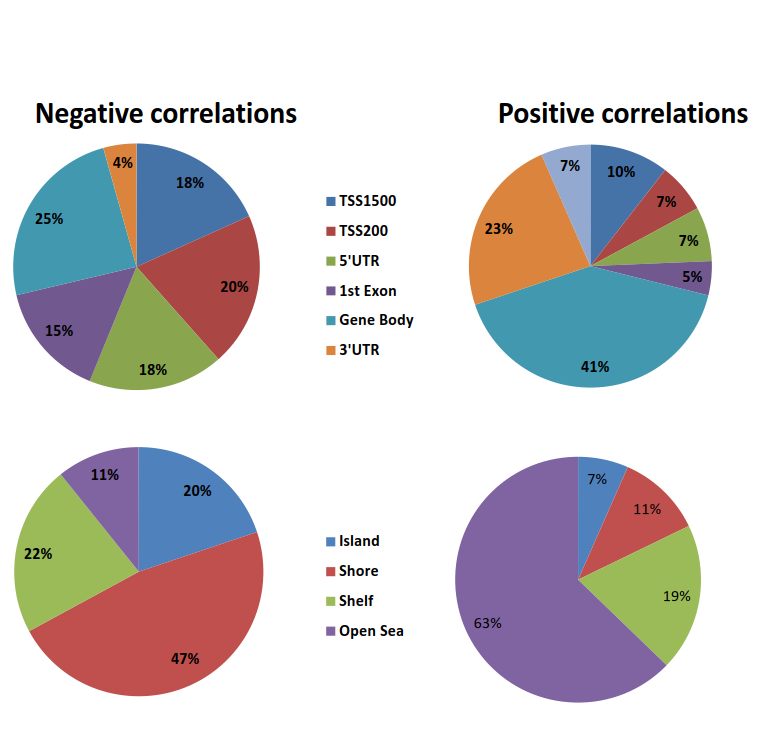


**Supplementary Figure S9:** Significant correlation between DNA methylation patterns in different gene regions and gene expression (BH adjusted P-value < 0.05). Pie chart plots show the distribution of negative and positive correlations corresponding to the subregions of genes. Distribution patterns are very different for the positive correlations compared to negative correlations.


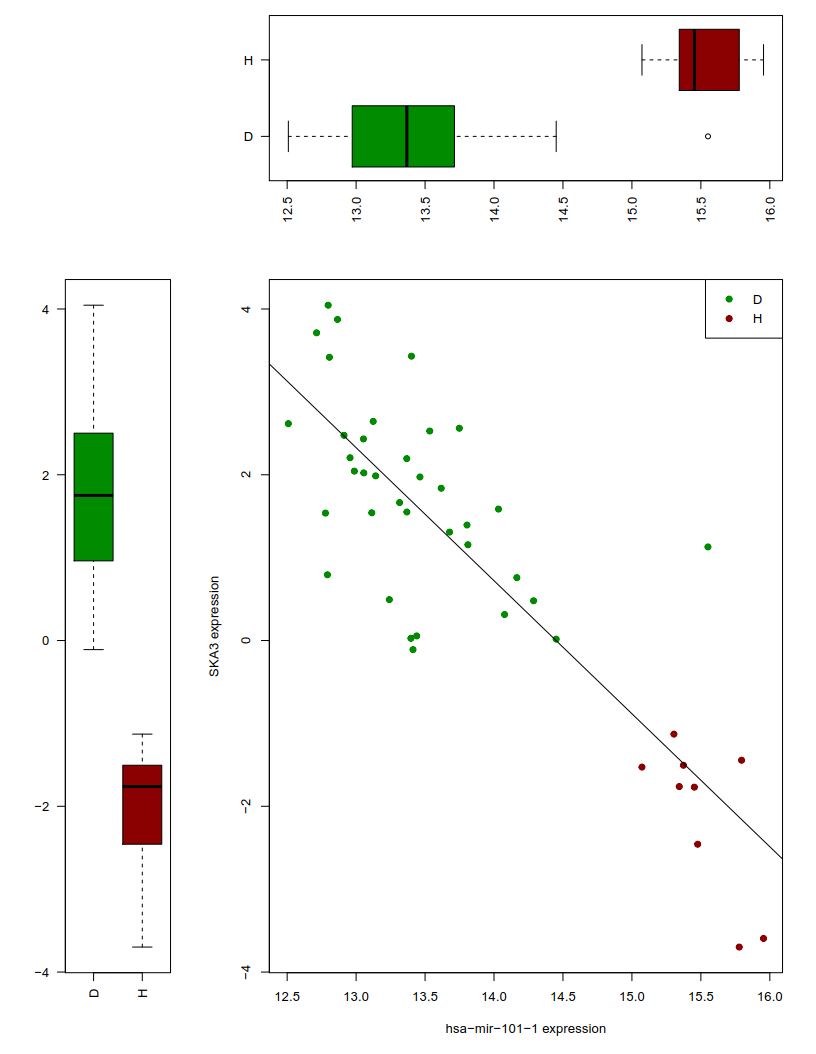


**Supplementary Figure S10:** An example of correlation between miRNA, hsa-mir-101 and its target genes’ expression (BH adjusted P-value < 0.05). In box plot D and H denotes disease and healthy respectively. This plot shows the distribution of a negative correlation between miRNA and gene expression. The gene expression and miRNA expression boxplots are on the left and top for gene and miRNA, respectively.


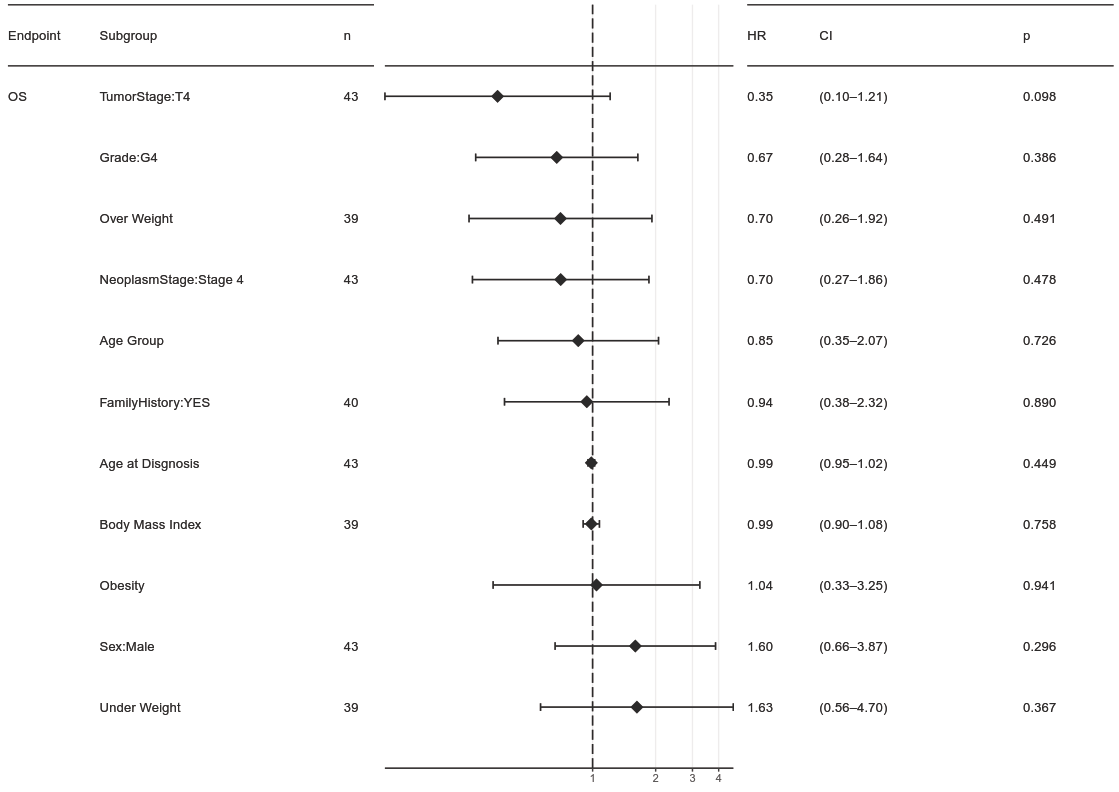


**Supplementary Figure S11:** Univariate Cox regression analysis. For subgroups, we make two groups of samples for the stage, grade, and neoplasm stage. We merge early-stage/grade/neoplasm-stage (1 and 2) in one group and late (3 and 4) in another group. For forest plot, we used R tool *survivalAnalysis*.

## Supplementary Tables

**Supplementary Table1:** Information of differentially methylated CpGs. Differential methylation information for lncRNAs and differential methylation frequency for each chromosome.

**Supplementary Table2:** Information of differentially methylated regions (DMRs) in CCA. Information of about DMRs overlap with VISTA super-enhancer, and DNase hypersensitive region.

**Supplementary Table3:** Information about differentially expressed genes and miRNAs.

**Supplementary Table4:** Results for pathway enrichment analysis for differentially expressed and differentially methylated genes. Table for the list of pathways for GMRCs and the top 5 subnetworks in GMRCs analysis.

**Supplementary Table5:** Table for the correlations based on eQTL analysis and, a correlation between genes and corresponding promoter CpGs. Table for the aggregate correlation of CpG in gene sub-region. Correlation between miRNA and gene expression.

**Supplementary Table6:** Correlation between gene and corresponding hypomethylated/hypomethylated distal enhancer CpGs. Transcription factor (TF) motif analysis results for hypermethylation and hypomethylation for distal enhancers and promoters.

**Supplementary Table7:** Survival analysis table for differentially expressed genes and miRNAs and, differentially methylated promoter CpGs.
